# Identification of novel canonical strigolactones produced by tomato

**DOI:** 10.3389/fpls.2022.1064378

**Published:** 2022-12-14

**Authors:** Takatoshi Wakabayashi, Daisuke Moriyama, Ayumi Miyamoto, Hironori Okamura, Nanami Shiotani, Nobuhiro Shimizu, Masaharu Mizutani, Hirosato Takikawa, Yukihiro Sugimoto

**Affiliations:** ^1^ Department of Agrobioscience, Graduate School of Agricultural Science, Kobe University, Kobe, Japan; ^2^ Faculty of Bioenvironmental Science, Kyoto University of Advanced Science, Kameoka, Japan; ^3^ Department of Applied Biological Chemistry, Graduate School of Agricultural and Life Sciences, The University of Tokyo, Tokyo, Japan

**Keywords:** biosynthesis, didehydroorobanchol, root parasitic weeds, strigolactone, structural determination

## Abstract

Canonical strigolactones (SLs), such as orobanchol, consist of a tricyclic lactone ring (ABC-ring) connected to a methylbutenolide (D-ring). Tomato plants have been reported to produce not only orobanchol but also various canonical SLs related to the orobanchol structure, including orobanchyl acetate, 7-hydroxyorobanchol isomers, 7-oxoorobanchol, and solanacol. In addition to these, structurally unidentified SL-like compounds known as didehydroorobanchol isomers (DDHs), whose molecular mass is 2 Da smaller than that of orobanchol, have been found. Although the SL biosynthetic pathway in tomato is partially characterized, structural elucidation of DDHs is required for a better understanding of the entire biosynthetic pathway. In this study, three novel canonical SLs with the same molecular mass as DDHs were identified in tomato root exudates. The first was 6,7-didehydroorobanchol, while the other two were not in the DDH category. These two SLs were designated phelipanchol and epiphelipanchol because they induced the germination of *Phelipanche ramosa*, a noxious root parasitic weed of tomato. We also proposed a putative biosynthetic pathway incorporating these novel SLs from orobanchol to solanacol.

## Introduction

Strigolactones (SLs) were originally discovered as germination stimulants for root parasitic weeds belonging to the *Striga*, *Orobanche*, and *Phelipanche* genera of the Orobanchaceae family (Cook et al., 1966; Aliche et al., 2020). These plant specialized metabolites also act as signaling molecules in the rhizosphere, inducing hyphal branching of arbuscular mycorrhizal fungi (Akiyama et al., 2005). SLs not only function as exogenous signaling molecules exuded by roots but also as endogenous growth regulators, attracting considerable attention as a novel class of phytohormones that regulate many aspects of plant development, including shoot branching/tillering and root architecture (Gomez-Roldan et al., 2008; Umehara et al., 2008; Aquino et al., 2021). SLs that contain a tricyclic lactone ring system (ABC-ring) are known as canonical SLs, in which the ABC-ring is connected to a methylbutenolide (D-ring) in the *R* configuration *via* an enol ether bridge, whereas non-canonical SLs have an incomplete ABC-ring system. Canonical SLs are classified into orobanchol- and strigol-type, in which the C-ring orientation is α and β, respectively. The naturally occurring SLs are of carotenoid origin. In the SL biosynthetic pathway, all-*trans*-β-carotene is converted to carlactone (CL), a common biosynthetic intermediate, by the sequential reaction catalyzed by carotenoid isomerase DWARF27 (D27), CAROTENOID CLEAVAGE DIOXYGENASE 7 (CCD7), and CCD8 enzymes (Alder et al., 2012). CL is oxidized to form carlactonoic acid (CLA) by the Arabidopsis cytochrome P450 monooxygenase MORE AXILLARY GROWTH 1 (MAX1/AtCYP711A1), and the conversion of CL to CLA is a conserved reaction in MAX1 homologs of various plant species (Abe et al., 2014; Seto et al., 2014; Yoneyama et al., 2018). The production of canonical SL was first characterized by rice MAX1 homologs. Os900/OsCYP711A2 catalyzes the closure of the BC-ring from CL to 4-deoxyorobanchol (4DO) *via* the intermediate CLA, and Os1400/OsCYP711A3 catalyzes the subsequent introduction of a hydroxy group at C-4 of 4DO to form orobanchol (Zhang et al., 2014).

**Figure 1:**
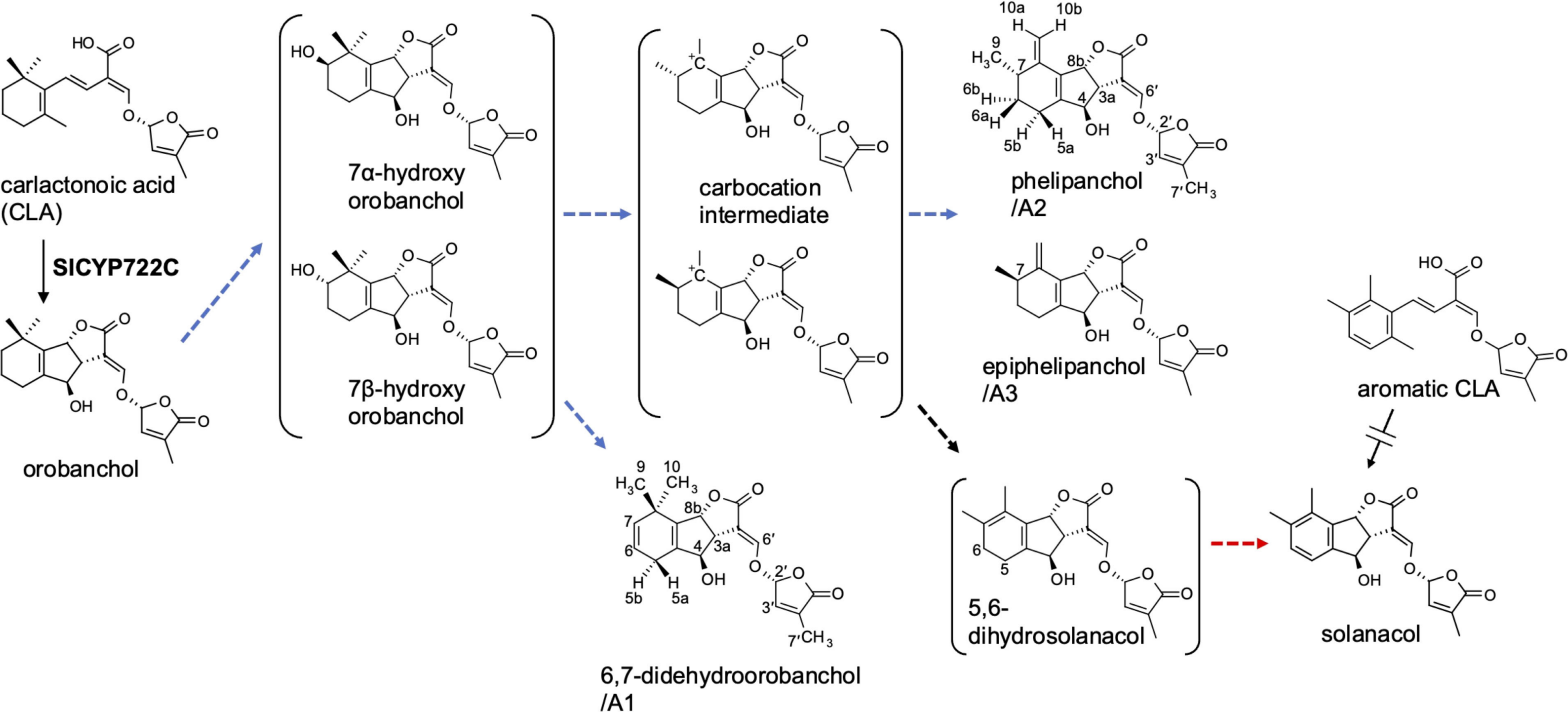
The proposed SL biosynthetic pathway from CLA to solanacol in tomato incorporating the novel SLs identified in this study. Blue and red dashed arrows indicate putative steps involving SlCYP712G1 and novel biosynthetic enzyme, respectively.

In SL production of tomato (*Solanum lycopersicum*), the CYP722C subfamily, which we identified as the key enzyme for canonical SL biosynthesis in dicot plants, is involved in the conversion of CLA to orobanchol without passing through 4DO (Wakabayashi et al., 2019; Wakabayashi et al., 2020) (**Figure 1**). Tomato exudes various canonical SLs in addition to orobanchol; orobanchyl acetate, 7-hydroxyorobanchol isomers, 7-oxoorobanchol, and solanacol have been identified by direct comparison with respective authentic samples (Koltai et al., 2010; Kohlen et al., 2013). Furthermore, LC-MS/MS analysis revealed at least three more structurally unidentified SL-like compounds, known as didehydroorobanchol isomers (DDHs) based on their molecular mass (Sato et al., 2003; López-Ráez et al., 2008; Kohlen et al., 2013). Although a previous study proposed that solanacol and DDHs are converted from orobanchol in tomato (Zhang et al., 2018), the structures of DDHs remain unknown. We must clarify the DDH structures and their structural relationship with other SLs to gain a better understanding of SL biosynthesis in tomato.

In this study, we report the isolation and structural determination of three novel SLs with the same molecular mass as DDHs from tomato root exudates. Two of the structures do not fall into the DDH category. These novel SLs induced the germination of *Phelipanche ramosa*, a troublesome root parasitic weed on tomato, implying that they are used as germination stimulants in the rhizosphere. Based on the determined structures, we proposed a biosynthetic pathway from orobanchol to solanacol *via* putative biosynthetic intermediates.

## Materials and methods

### General procedures

^1^H and ^13^C NMR spectra were recorded in C_6_D_6_ on a Bruker Biospin AC400M spectrometer (400 MHz for ^1^H and 100 MHz for ^13^C). Standard pulse sequence and phase cycling were used for HSQC, HMBC, COSY, and NOESY spectra analyses. Chemical shifts were referenced to C_6_D_6_ (*δ*_H_ 7.16, *δ*_C_ 128.06). UV and CD spectra were obtained using a V-630 spectrophotometer and a J-805 spectropolarimeter (JASCO, Tokyo, Japan), respectively. High resolution mass spectra were recorded using an Orbitrap Exploris 240 mass spectrometer equipped with an ESI source (HR-ESI-MS), connected to an Ultimate 3000 HPLC system (Thermo Fisher Scientific). LC-MS/MS analyses were performed using an LC-MS/MS system (Waters) comprising Acquity UPLC H-Class and an Acquity TQD tandem mass spectrometer. Data acquisition and analyses were performed using MassLynx 4.1 software (Waters).

### Chemicals

*Rac*-orobanchol was prepared as reported previously (Matsui et al., 1999). Aromatic CLA was synthesized from 2,3,6-trimethylphenol based on the previously reported methods (Wakabayashi et al., 2019; Shiotani et al., 2021). The **Supplementary Methods** provide more information.

### Collection of root exudates from tomato hydroponic solution

Tomato cultivars obtained from a local market in Japan were grown hydroponically according to the following procedure. Surface sterilized tomato seeds were sown on MS agar plates and grown for 11 days at 23°C with a 16-h light/8-h dark photoperiod. Then, the plants were transferred to test tubes containing 50 ml half-strength Hoagland nutrient solution without phosphate. After three days, the nutrient solution was replaced with a fresh one and each plant was grown for a week. Each filtrate was subjected to purification with a solid phase extraction using an Oasis HLB cartridge (Waters, USA) as described previously (Moriyama et al., 2022). An aliquot (5 µL) of the extract dissolved in 100 µL acetonitrile was subjected to an LC-MS/MS analysis.

The selected tomato cultivar, Yellow-Pico, tomato seedlings were grown hydroponically in a tank (245×365×140 mm) filled with phosphate-limited half-strength Hoagland nutrient solution (phosphorus concentration reduced to one-tenth of half-strength Hoagland nutrient solution) at 25°C with a 16-h light/8-h dark photoperiod. The solution was continuously circulated using a pump, and root exudates of 3–16 week old plants were adsorbed on XAD-4 (2.1 L in total) as described previously (Ueno et al., 2014). The XAD-4, replaced every week with a fresh one, was soaked in acetone (16.8 L in total). SLs were extracted by sonication and filtered. After evaporating the acetone, the residual aqueous solution was treated three times with the same volume of EtOAc. The organic layer was dried over Na_2_SO_4_ and concentrated in vacuo.

### Isolation of 6,7-didehydroorobanchol/A1, phelipanchol/A2, and epiphelipanchol/A3

The crude extracts of tomato root exudate (4 g) were subjected to silica gel column chromatography (Silica Gel 60, spherical, neutral 120 g). Stepwise elution with a solvent system of *n*-hexane–EtOAc (100:0–0:100, 10% step, 827 mL each) produced 11 fractions (Frs. 1–11). A mixture of 6,7-didehydroorobanchol/A1, phelipanchol/A2, and epiphelipanchol/A3, eluted mostly in Fr. 8 (*n*-hexane–EtOAc, 30:70, v/v), was further purified using reversed-phase HPLC on a COSMOSIL 5C18-MS-II column (250 × 10 mm i.d., 5 µm) (Nacalai Tesque Inc., Kyoto, Japan) with a CH_3_CN/H_2_O (30:70, v/v) isocratic system at 5.0 mL/min flow rate. Eluents were monitored at 254 nm for three peaks at 39, 40.5, and 41.5 min, yielding 6,7-didehydroorobanchol/A1 (140 μg) phelipanchol/A2 (930 μg) and epiphelipanchol/A3 (130 μg), respectively.

**Tables 1**, **2**; **Supplementary Tables S1, S2** and **Supplementary Figures S5-S16** show NMR spectroscopic data on 6,7-didehydroorobanchol/A1, phelipanchol/A2, and epiphelipanchol/A3. HR-ESI-MS *m/z* calcd for [C_19_H_20_O_6_ + H]^+^: 345.13326, found 345.1331 (6,7-didehydroorobanchol/A1); 345.1329 (phelipanchol/A2); and 345.1330 (epiphelipanchol/A3). Mass spectra (**Supplementary Figure S1**). CD (6,7-didehydroorobanchol/A1: CH_3_CN, *c* 0.000016) *λ*_ext_ (Δϵ) nm: 227 (6.05); (phelipanchol/A2: CH_3_CN, *c* 0.000017) *λ*_ext_ (Δϵ) nm: 223 (4.78); (epiphelipanchol/A3: CH_3_CN, *c* 0.000013) *λ*_ext_ (Δϵ) nm: 225 (7.10) (**Supplementary Figure S2**).

**Table 1:** ^1^H NMR spectroscopic data of A1, A2 and A3.

No.	A1 (C_6_D_6_)δ ^1^H (mult., *J* Hz)	A2 (C_6_D_6_)δ ^1^H (mult., *J* Hz)	A3 (C_6_D_6_)δ ^1^H (mult., *J* Hz)	orobanchol (C_6_D_6_)*δ ^1^H (mult., *J* Hz)
2				
3				
3a	3.17 (*ddd*, 2.0, 2.5, 7.3)	3.17 (*ddd*, 2.1, 2.5, 7.3)	3.18 (*ddd*, 2.0, 2.5, 7.3)	3.15 (*ddd*, 2.0, 2.6, 7.3)
4	4.26 (*br*, *s*)	4.32 (*br*, *s*)	4.33 (*br*, *s*)	4.28 (*br d*, 6.7)
4a				
5a	2.47 (*dddd*, 1.0, 1.7, 3.0, 22.6)	1.94 (*dddd*, 0.5, 5.5, 7.9, 18.2)	1.98 (*dddd*, 1.9, 5.3, 5.6, 18.1)	1.81 (*m*)
5b	2.12 (*dddd*, 1.5, 2.3, 3.2, 22.6)	1.69 (*dddd*, 1.2, 5.3, 5.9, 18.2)	1.60 (*ddd*, 5.6, 8.3, 18.1)	1.50 (*td*, 5.4, 12.4)
6a	5.40 (*ddd* 3.0, 3.2, 9.9)	1.21 (*dddd*, 5.3, 7.9, 8.5, 12.9)	1.17 (*dddd*, 5.3, 8.3, 8.9, 12,9)	1.14-1.20 (*m*)
6b		1.44 (*dddd*, 4.0, 5.5, 5.9, 12.9)	1.45 (*dddd*, 3.9, 5.6, 5.6, 12.9)	
7	5.34 (*ddd*, 1.5, 1.7, 9.9)	2.17 (*ddq*, 4,0, 8.5, 6.8)	2.15 (*ddq*, 3.9, 8.9, 6.8)	1.21-1.44 (*m*)
8				
8a				
8b	5.22 (*dddd*, 1.0, 2.0, 2.3, 7.3)	5.24 (*dddd*, 0.5, 1.2, 2.2, 7.3)	5.29 (*ddd*, 1.9, 3.0, 7.3)	5.18 (*ddd*, 1.7, 2.2, 7.3)
9	1.06 (*s*)	0.96 (*d*, 6.8)	0.97 (*d*, 6.8)	0.97 (*s*)
10a	1.30 (*s*)	5.03 (*br*, *s*)	5.03 (*br*, *s*)	1.18 (*s*)
10b		5.38 (*br*, *s*)	5.35 (*br*, *s*)	
2’	5.00 (*dq*, 1.8, 1.5)	5.02 (*dq*, 1.5, 1.5)	4.98 (*dq*, 1.7, 1.5)	5.02 (*br*, *s*)
3’	5.66 (*dq*, 1.8, 1.5)	5.67 (*dq*, 1.5, 1.5)	5.66 (*dq*, 1.7, 1.5)	5.66 (*t*, 1.6)
4’				
5’				
6’	7.37 (*d*, 2.5)	7.36 (*d*, 2.5)	7.35 (*br*, *s*)	7.37 (*d*, 2.6)
7’	1.33 (*dd*, 1.5, 1.5)	1.33 (*dd*, 1.5, 1.5)	1.33 (*dd*, 1.5, 1.5)	1.32 (*t*, 1.6)
4-OH		1.11 (*s*)	1.07 (*d*, 5.5)	1.07 (*d*, 6.7)

*Tokunaga et al. (2015).

**Table 2:** NMR spectroscopic data of A2.

No.	δ ^1^H (mult., *J* Hz)	δ ^13^C	^1^H-^1^H COSY	HMBC	NOESY
2		170.1			
3		111.5			
3a	3.17 (*ddd*, 2.0, 2.5, 7.3)	49.0	H-4, H-8b, H-6’	C-2, C-3, C-4, C-4a, C-6’	H-4, H-8b
4	4.26 (*br*, *s*)	83.1	H-3a, H-8b, 4-OH		H-3a, H-5b
4a		136.4			
5a	2.47 (*dddd*, 1.0, 1.7, 3.0, 22.6)	34.2	H-5b, H-6a, H-6b, H-8b	C-4a, C-6, C-8, C-8a	H-5b, H-6a, H-6b, H-7
5b	2.12 (*dddd*, 1.5, 2.3, 3.2, 22.6)		H-5a, H-6a, H-6b	C-4a, C-6, C-8, C-8a	H-4, H-5a, H-6a, H-6b, H-9
6a	5.40 (*ddd* 3.0, 3.2, 9.9)	30.5	H-5a, H-5b, H-6b, H-7	C-5, C-7, C-8, C-8a	H-5a, H-5b, H-6b, H-7, H-9
6b			H-5a, H-5b, H-6a, H-7	C-5, C-7, C-8, C-8a, C-9	H-5a, H-5b, H-6a, H-7, H-9
7	5.34 (*ddd*, 1.5, 1.7, 9.9)	22.2	H-6a, H-6b, H-9, H-10a, H-10b		H-5a, H-6a, H-6b, H-9, H-10a
8		144.2			
8a		146.6			
8b	5.22 (*dddd*, 1.0, 2.0, 2.3, 7.3)	84.2	H-3a, H-4, H-5a	C-2, C-8a	H-3a, H-10b
9	1.06 (*s*)	18.9	H-7	C-5, C-6, C-8	H-5b, H-6a, H-6b, H-7, H-10a
10a	1.30 (*s*)	110.9	H-7, H-10b	C-4a, C-5, C-7, C-8	H-7, H-9, H-10b
10b			H-7, H-10a	C-4a, C-5, C-7, C-8	H-8b, H-10a
2’	5.00 (*dq*, 1.8, 1.5)	100.7	H-3’, H-7’	C-4’, C-5’, C-6’	H-3’, H-6’
3’	5.66 (*dq*, 1.8, 1.5)	140.6	H-2’, H-7’	C-2’, C-5’	H-2’, H-7’
4’		135.2			
5’		169.8			
6’	7.37 (*d*, 2.5)	151.4	H-3a	C-2, C-3, C-3a, C-2’	H-2’
7’	1.33 (*dd*, 1.5, 1.5)	10.2	H-2’, H-3’	C-2’, C-3’, C-4’, C-5’	H-3’
4-OH			H-4		

### SL analysis

The SLs were analyzed using LC-MS/MS under analytical conditions as previously described (Wakabayashi et al., 2019). Chromatographic separation was performed with an ODS column (COSMOSIL 2.5C18-MS-II, 100 × 2.0 mm i.d., 2.5 µm; Nacalai Tesque) at a column oven temperature of 30°C. The elution was performed in a linear gradient system of MeOH–H_2_O with 0.1% formic acid (50:50–100:0 in 20 min) at a flow rate of 0.2 mL min^−1^. The MRM transitions selected were at *m/z* 347.1 > 233.1 for orobanchol, 345.2 > 97 for DDHs, and 343.1 > 97 for solanacol, in the positive ESI mode. The cone voltage and the collision energy were 25 V and 18 eV, respectively.

### Germination assay

The germination assay was conducted as reported previously (Sugimoto et al., 2003). Seeds of *P. ramosa* and *S. hermonthica* were collected from mature plants of tomato in Khatroum and sorghum in Gedarif, Sudan, respectively. The surface sterilized seeds were pretreated (conditioned) for 10 days at 30°C for *S. hermonthica*, and 23°C for *P. ramosa* on 8-mm glass-fiber filter paper disks (ca. 50 seeds each) placed on distilled water-saturated filter paper. Aliquots (20 μL) of diluted test solutions were assayed by applying them to conditioned seeds on 8 mm disks. The treated seeds were incubated at the same temperature as that used for the conditioning and were microscopically examined after 1 day (*S. hermonthica*) and 5 days (*P. ramosa*).

## Results

### Isolation and structure determination of three novel SLs from tomato root exudates

In a previous study, we generated *SlCYP722C*- knocked out (KO) tomato plants (cv. Micro-Tom) by CRISPR/Cas9-mediated genome editing to investigate its function in planta. Orobanchol and solanacol levels in root exudates of *SlCYP722C*-KO tomato plants decreased to below the detection limit, but CLA, a substrate of the SlCYP722C enzyme, was abundant (Wakabayashi et al., 2019). At least three DDHs were found in the tomato hydroponic solutions (Kohlen et al., 2013; Zhang et al., 2018). In our LC-MS/MS analytical conditions with an ODS column, SL-like compounds with the same molecular mass as DDHs were aggregated as a single peak on the chromatogram. However, the peak disappeared in the *SlCYP722C*-KO tomato exudates, indicating that the SL-like compounds are metabolites downstream of orobanchol (Wakabayashi et al., 2019) (**Supplementary Figure S3**). We qualitatively and quantitatively analyzed SLs in root exudates of more than 20 tomato cultivars available in the local market in Japan. As a result, the cultivar Yellow-Pico (YP) was selected as the highest producer of the SL-like compounds for further isolation and structural elucidation (**Supplementary Figure S4**).

**Figure 2:**
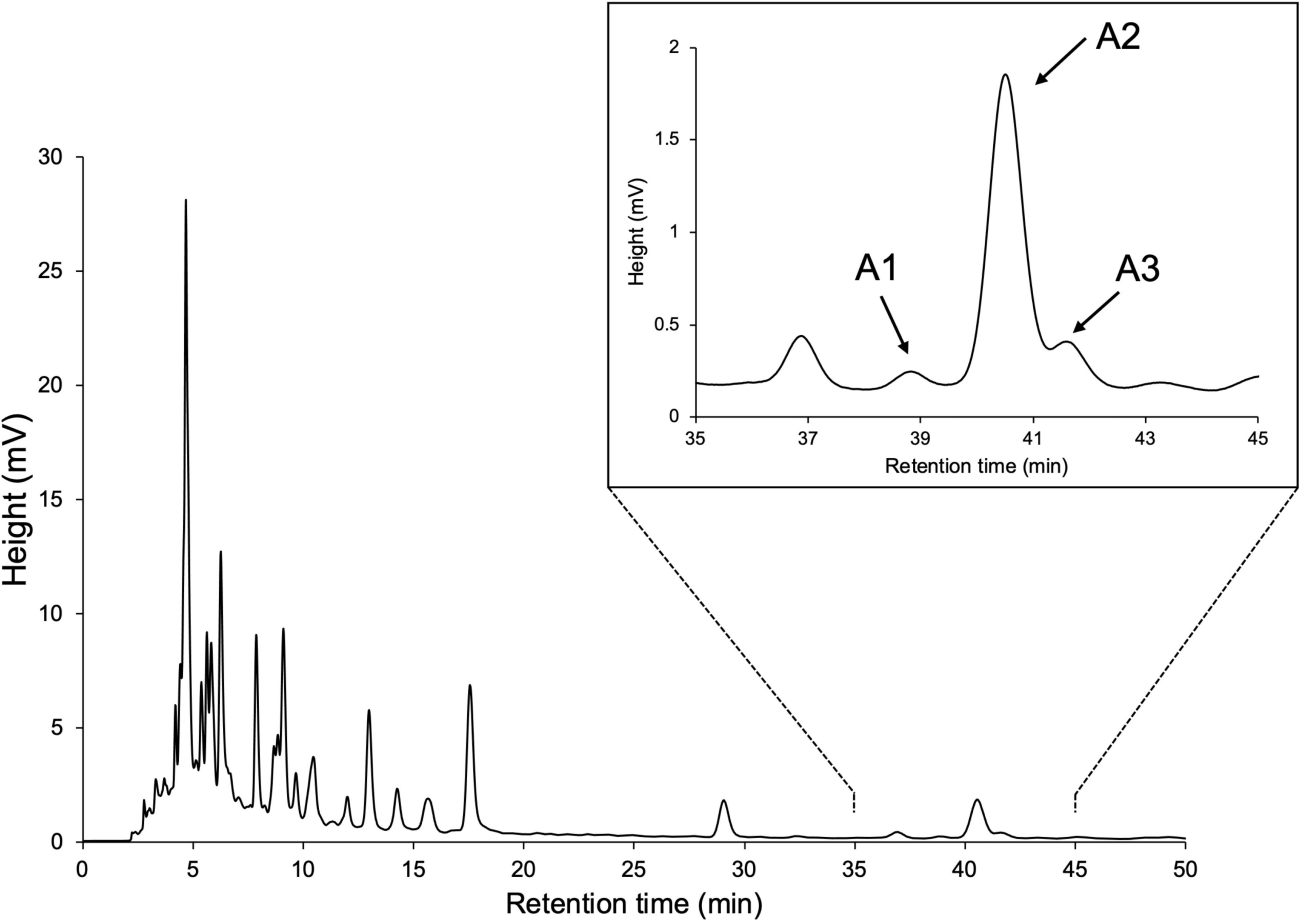
HPLC separation of SL-like compounds (A1, A2, and A3) with the same molecular mass as DDHs.

YP was grown hydroponically in a phosphate-limited half-strength Hoagland nutrient solution, and root exudates were obtained by absorption on XAD-4 as previously described (Ueno et al., 2014; Moriyama et al., 2022). Purification of YP root exudates by silica gel column chromatography guided by LC-MS/MS analysis resulted in enriched fractions of SL-like compounds eluted with 30% EtOAc in *n*-hexane. We separated the SL-like compounds using HPLC on a 25-cm long ODS column, with three SL-like compounds eluting at 39, 40.5, and 41.5 min (**Figure 2**). These novel SLs were tentatively called A1, A2 and A3 in the order of their elution from reversed-phase chromatography because their identity to the previously reported DDHs (Kohlen et al., 2013; Zhang et al., 2018) was unknown. The molecular formula of A1, A2 and A3 was established to be C_19_H_20_O_6_ based on proton adduct ion [M + H]^+^ at *m/z* 345.1331, 345.1329, and 345.1330, respectively, obtained using HR-ESI-MS, which is consistent with that of DDH (calcd. for [C_19_H_20_O_6_ + H]^+^, 345.13326). The mass spectra of A1, A2, and A3 were almost identical and exhibited major common fragment ions at *m/z* 97, indicating that they have the D-ring of SL (**Supplementary Figure S1**). A1, A2, and A3 exhibited slightly different CD spectra, with A2 and A3 having a positive cotton effect around 250 nm, which is not observed in A1 (**Supplementary Figure S2**).

**Table 1** lists ^1^H NMR spectroscopic data of A1, A2, and A3 aided with 2D NMR experiments, including ^1^H-^1^H COSY, and NOESY. Compared with the orobanchol data (Tokunaga et al., 2015), remarkable differences were observed in methylene protons in the A-ring in A1. ^1^H NMR spectra of A2 and A3 closely resembled each other. Typical canonical SLs have 19 carbons with two geminal methyl groups at C-8 in the A-ring and an olefin methyl group at C-4′ in the D-ring. In contrast to A1 and orobanchol, A2 and A3 have only one methyl group in the A-ring. Instead, two mutually coupled broad ^1^H singlets corresponding to a methylidene group were observed.

**Figure 3:**
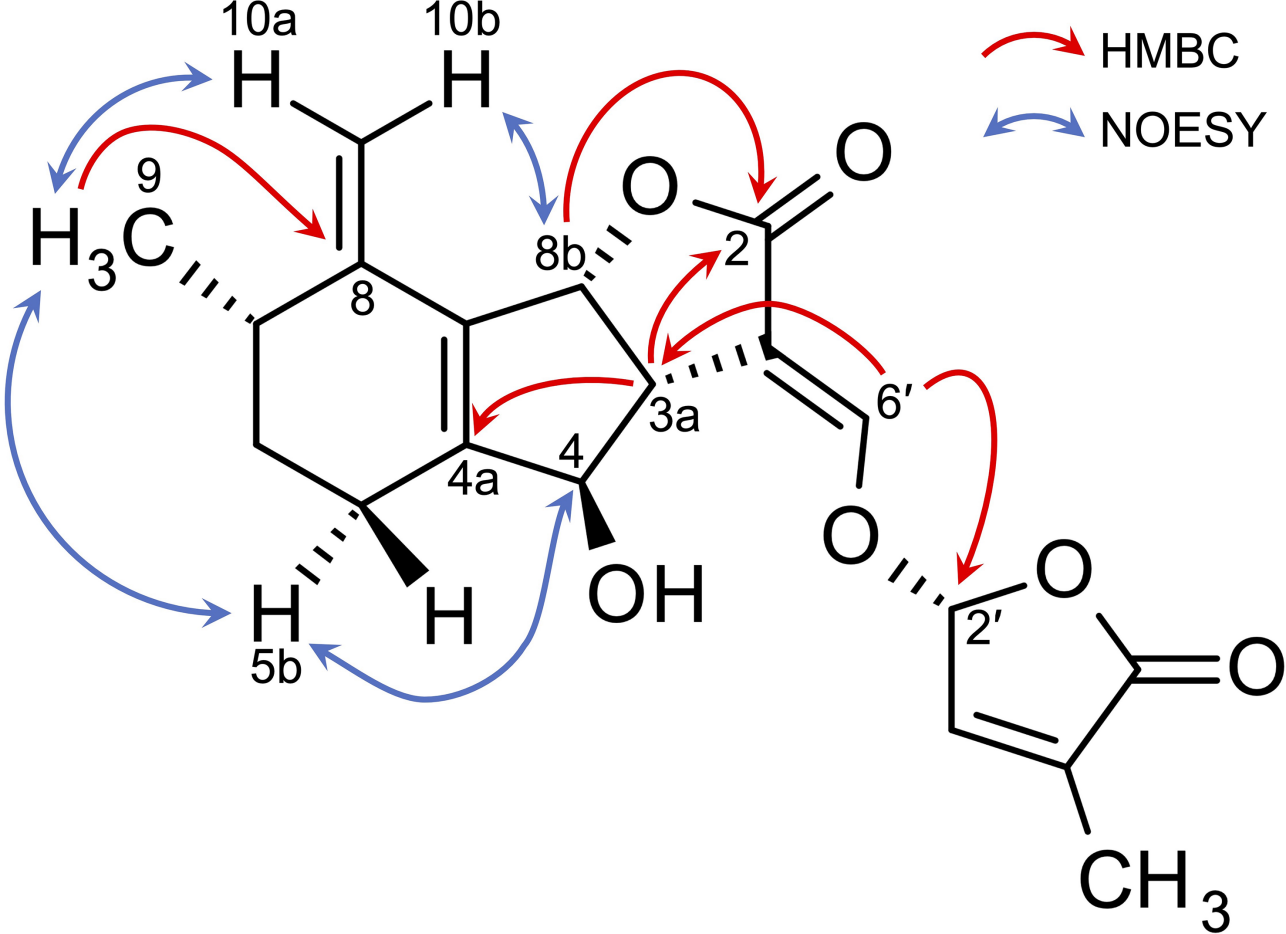
Key HMBC and NOESY correlations of phelipanchol/A2.

**Table 2** shows ^13^C NMR, COSY, HMBC, and NOESY spectroscopic data from A2, indicating two methyl groups (H-9 and H-7′), two methylene groups (H-5a,b, and H6a,b), five methine groups (H-3a, H-4, H-7, H-8b, and H-2′), two olefin protons (H-3′ and H-6′) and two methylidene protons (H-10a,b). The ^1^H-^1^H COSY and HMBC correlations from H-2′, H-3′, H-6′, and H-7′ together with the mass fragment ions at *m/z* 97 and 231 showed the conserved enol ether bridged methylbutenolide (the D-ring). The ^1^H-NMR spectroscopic data exhibited mutually coupled methine protons (H-3a at δ3.17 and H-8b at δ5.24), which are characteristic signals observed in canonical SLs. A broad methine singlet at δ4.32, corresponding to H-4 in orobanchol, was also observed. The HMBC experiment confirmed that the H-3a signal correlated with the C-2, C-3, C-4, C-4a, and C-6′ resonances, and the H-8b signal correlated with the C-2 and C-8a resonances. Correlations of an olefin proton (H-6′ at δ7.36) with the C-2, C-3, C-3a, and C-2′ resonances were also observed. These results indicated a γ-lactone moiety and cyclopentene moiety fused to form the BC-ring. Protons H5a,b, and H6a,b were mutually coupled. The ^1^H-^1^H COSY correlations of H-7 with protons H-6a,b, H-9, and H-10a,b were observed. The HMBC correlations of H-5a,b to C-4a, C-6, C-8, and C-8a, those of H-6a,b to C-5, C-7, C-8, C-8a, and C-9, those of H-9 to C-5, C-6, C-8, and those of H-10a,b to C-4a, C-5, C-7, and C-8 revealed a cyclohexene A-ring with a methyl group at C-7 and a methylidene group at C-8. H-10a and H-10b were distinguishable by the NOESY correlations of H-10a with H-7 and H-9, and that of H10b with H-8b. Thus, the unique structure of A2 having a methyl group and a methylidene group at C-7 and C-8 in the A-ring, respectively, was established (**Figures 1**, **3**). The NOESY correlations of H-5b with H-4 and H-9 indicated that the absolute configuration at C-7 is *S* (**Figure 3**).

The chemical shift and coupling patterns of A3’s ^1^H NMR spectrum were very similar to those of A2. Little but critical difference was observed in the chemical shift of H-5b, which was shifted in an up-filed by 0.09 ppm compared with that of A2 (**Table 1**). A comprehensive ^13^C NMR spectrum of A3 was not recorded because of the limited amount. However, significant correlations were observed in COSY and NOESY spectroscopic data and all the proton signals were assigned with reference to those in A2. The NOESY correlations of H-5a and H-9 (**Supplementary Table S1**) strongly indicate that A3 is the epimer of A2 at C-7 (**Figure 1**).

The ^1^H NMR spectroscopic data of A1 revealed three methyl groups, which are typical of canonical SLs (**Table 1**). Remarkable differences between A1 and orobanchol were observed in the methylene proton signals in the A-ring. The methylene protons at δ2.12 and δ2.47 in A1 resonated at a downfield compared with those in orobanchol. Mutually coupled olefinic protons correlated with the methylene protons were observed at δ5.40 and δ5.34 in the ^1^H-^1^H COSY spectrum. The coupling constants of the methylene protons with the former (3.0 and 3.2 Hz) were larger than those with the latter (1.5 and 1.7 Hz). The NOESY correlations of the methine proton at δ5.22 (H-8b) with the methyl protons at δ1.06 (H-9) and those of the olefinic proton at δ5.34 (H-7) with methyl protons at δ1.06 (H-9) and δ1.30 (H-10) were observed (**Supplementary Table S2**). These data indicate a double bond between C-6 and C-7 in the A-ring of A1. Thus, the novel SL is identified as 6,7-didehydroorobanchol (**Figure 1**).

### The germination-inducing activities of novel SLs for root parasitic weeds

**Figure 4:**
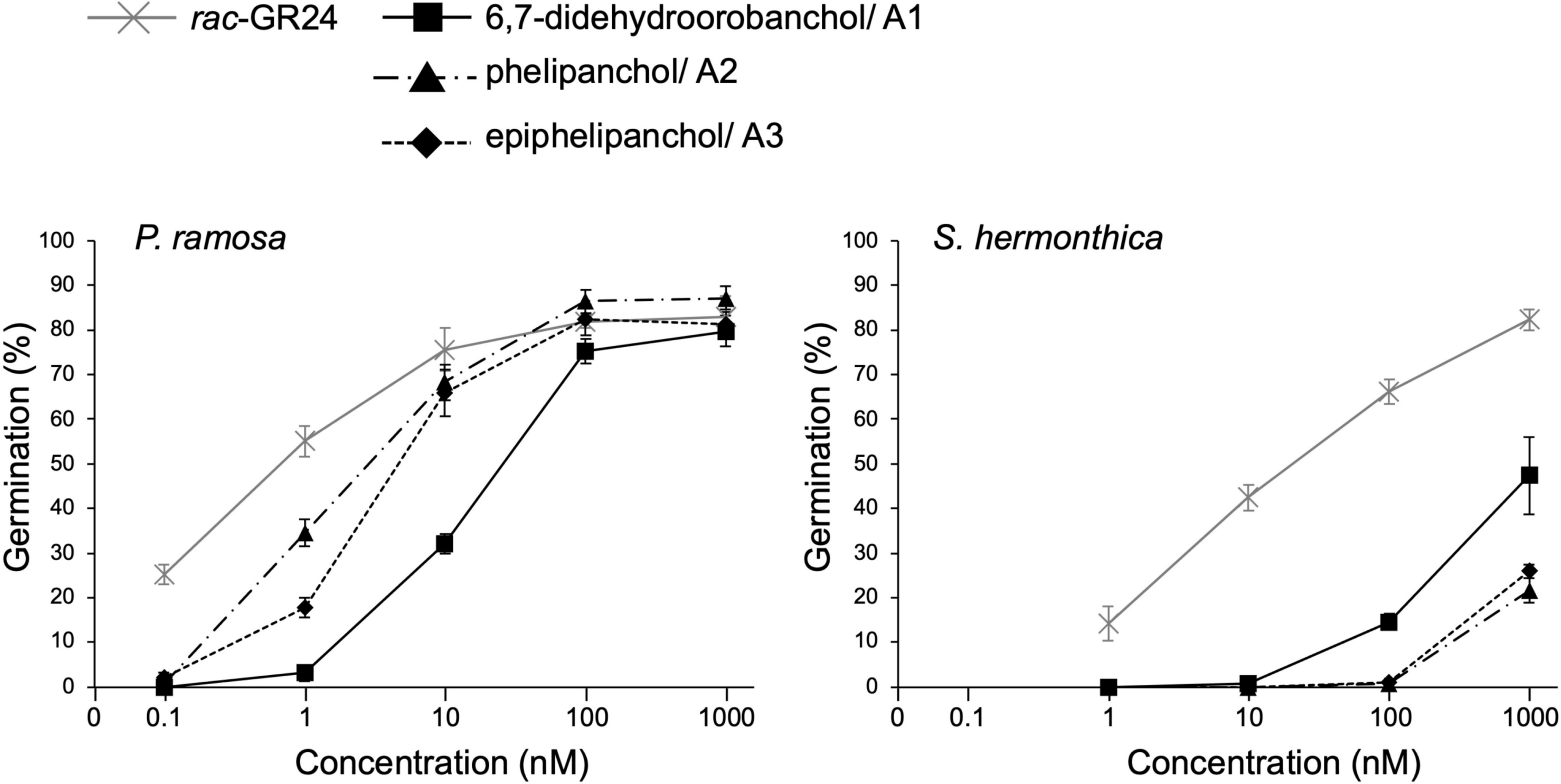
Germination-inducing activities of 6,7-didehydroorobanchol/A1, phelipanchol/A2, and epiphelipanchol/A3 isolated from tomato and synthetic SL analog *rac*-GR24 for *P. ramosa* and *S. hermonthica*. Data are presented as means ± SE (*n* = 3).

**Figure 4** shows the germination-inducing activities of 6,7-didehydroorobanchol/A1, A2, and A3 for *P. ramosa* and *S. hermonthica*. All of these SLs elicited the germination of *P. ramosa*, which is causing damage to tomato with significant yield losses (Parker, 2013). The 6,7-didehydroorobanchol/A1 showed lower germination-inducing activity than A2 and A3, and the different activity may reflect structural differences in the A-ring. A2 and A3, which structurally do not fall into the DDH category, were named phelipanchol and epiphelipanchol, respectively, because of their potent germination-inducing activities for the germination of *P. romosa*.

## Discussion

### Identification of phelipanchol, epiphelipanchol, and 6,7-didehydroorobanchol as novel tomato SLs with the same molecular mass as DDHs

Three novel canonical SLs with the same molecular mass as DDHs, 6,7-didehydroorobanchol/A1, phelipanchol/A2, and epiphelipanchol/A3, were isolated from root exudates of tomato. Based on detailed analyses of NMR, mass, and CD spectra, their structures were established (**Figure 1**). Note that 6,7-didehydroorobanchol/A1 is a DDH isomer but phelipanchol/A2 and epiphelipanchol/A3 do not fall into the category of DDH isomers. Solanacol was reported to be effective in inducing germination of *P. ramosa* seeds (Xie et al., 2007). These novel SLs induced germination of root parasitic weeds and showed potent germination-inducing activity for *P. ramose* similar to that of solanacol, but weak activity for *S. hermonthica* (**Figure 4**), which was consistent with the finding that orobanchol-type SLs showed weak germination-inducing activity for *S. hermonthica* (Ueno et al., 2011; Nomura et al., 2013; Wakabayashi et al., 2022). Root parasitic weeds are likely to use these SLs as germination stimulants in the tomato rhizosphere. Furthermore, natural canonical SLs are known to be highly active in inducing hyphal branching in arbuscular mycorrhizal fungi (Akiyama et al., 2010), indicating that these SLs may function similarly.

### Biosynthesis pathway from orobanchol to solanacol in tomato

The conversion of orobanchol to solanacol and DDH(s) was demonstrated in tomato (Zhang et al., 2018) in accordance with our observation that these SLs decreased to below the detection limit in root exudates of *SlCYP722C*-KO tomato plants (Wakabayashi et al., 2019) (**Supplementary Figure S3**). In addition, 7-α-hydroxyorobanchol and 7-β-hydroxyorobanchol were previously isolated from cucumber (*Cucumis sativus*) as orobanchol-related compounds (Khetkam et al., 2014) and identified in tomato root exudates (Kohlen et al., 2013). Considering the structures of novel SLs elucidated in this study and the possible presence of hydroxyorobanchol isomers, a plausible biosynthetic pathway from orobanchol to solanacol can be proposed (**Figure 1**). A hydroxy group is introduced into orobanchol at C-7, probably in α and β orientations. Dehydration of the 7-hydroxyorobanchol isomers could yield 6,7-didehydroorobanchol/A1 irrespective of the orientation of the hydroxyl group at C-7. Alternatively, introduction of a hydroxy group at C-6, followed by dehydration, could also yield 6,7-didehydroorobanchol as described by Wang et al. (2022). The migration of a methyl group at C-8 of the 7-hydroxyorobanchol isomers to C-7 assisted by elimination of the hydroxy group could result in carbocation intermediates. The formation of a double bond in the exocyclic position at C-8 of the intermediates could result in phelipanchol/A2 and its 7-epimer epiphelipanchol/A3. The orientation of the methyl group at C-7 is assumed to depend on the migration mechanism of the methyl group at C-8 in the 7-hydroxyorobanchol isomers. When the double bond is formed in the endocyclic position in the A-ring of the carbocation intermediates, they lose chirality at C-7 to yield 5,6-dihydrosolanacol. In this study, 5,6-dihydrosolanacol could not be isolated and is suspected to be present only in trace amounts as a biosynthetic intermediate because of its chemical instability. Furthermore, SlCYP712G1 in tomato was recently identified as an enzyme responsible for the conversion of orobanchol to three DDH structures, which remain unknown but include 5,6-dihydrosolanacol as a putative structure (Wang et al., 2022). Different from our proposed biosynthetic pathway, the authors hypothesized that DDHs and solanacol are derived from one of the hydroxyorobanchol isomers. The structural determination of SlCYP712G1 reaction products, including DDHs and hydroxyorobanchol isomers, is a future challenge, but if SlCYP712G1 is responsible for the reactions to the three novel SLs identified in this study, it would catalyze the above series of reactions (**Figure 1**).

In addition to the above proposed biosynthetic pathways, we hypothesized a biosynthetic route to solanacol from the aromatic A-ring analog of CLA (aromatic CLA), which has the same structure in the A-ring as that of solanacol. If the A-ring of CLA is aromatized before the BC-ring formation, SlCYP722C may convert aromatic CLA to solanacol (**Figure 1**). To examine this hypothesis, aromatic CLA was synthesized and used as a substrate in an enzyme reaction for SlCYP722C *in vitro*. However, the enzyme reaction of SlCYP722C with aromatic CLA did not result in the formation of solanacol (data not shown). Therefore, it is likely that the A-ring of solanacol is formed after the BC-ring closure, probably *via* 5,6-dihydrosolanacol (**Figure 1**).

Future studies are required to elucidate the biosynthetic pathway from orobanchol to solanacol and the role of the structurally diverse SLs produced by tomato in the rhizosphere. This study provides new insights that will contribute to the entire understanding of the SL biosynthetic pathway in tomato.

## Data availability statement

The original contributions presented in the study are included in the article/**Supplementary Material**. Further inquiries can be directed to the corresponding author.

## Author contributions

TW and YS contributed to conception and design of the study. DM and AM performed the experiments. HO, NaS, and HT synthesized compounds. TW, DM, NoS, MM, and YS analyzed the data. TW and YS wrote the manuscript with assistance from all authors. All authors contributed to manuscript revision, read, and approved the submitted version.
