# Corrigendum: Identification of novel canonical strigolactones produced by tomato

**DOI:** 10.3389/fpls.2023.1151993

**Published:** 2023-02-13

**Authors:** Takatoshi Wakabayashi, Daisuke Moriyama, Ayumi Miyamoto, Hironori Okamura, Nanami Shiotani, Nobuhiro Shimizu, Masaharu Mizutani, Hirosato Takikawa, Yukihiro Sugimoto

**Affiliations:** ^1^ Department of Agrobioscience, Graduate School of Agricultural Science, Kobe University, Kobe, Japan; ^2^ Faculty of Bioenvironmental Science, Kyoto University of Advanced Science, Kameoka, Japan; ^3^ Department of Applied Biological Chemistry, Graduate School of Agricultural and Life Sciences, The University of Tokyo, Tokyo, Japan

**Keywords:** biosynthesis, didehydroorobanchol, root parasitic weeds, strigolactone, structural determination

**Error in Table**

In the published article, there was an error in **Table 2** as published. In **Table 2**, the chemical shift of compound A2’s ^1^H NMR spectrum should be listed, but it was incorrectly listed as that of compound A1.

The corrected **Table 2** and its caption “NMR spectroscopic data of A2” appear below.

**Table 2:** NMR spectroscopic data of A2.

No.	δ ^1^H (mult., *J* Hz)	δ ^13^C	^1^H-^1^H COSY	HMBC	NOESY
2		170.1			
3		111.5			
3a	3.17 (*ddd*, 2.1, 2.5, 7.3)	49.0	H-4, H-8b, H-6’	C-2, C-3, C-4, C-4a, C-6’	H-4, H-8b
4	4.32 (*br*, *s*)	83.1	H-3a, H-8b, 4-OH		H-3a, H-5b
4a		136.4			
5a	1.94 (*dddd*, 0.5, 5.5, 7.9, 18.2)	34.2	H-5b, H-6a, H-6b, H-8b	C-4a, C-6, C-8, C-8a	H-5b, H-6a, H-6b, H-7
5b	1.69 (*dddd*, 1.2, 5.3, 5.9, 18.2)		H-5a, H-6a, H-6b	C-4a, C-6, C-8, C-8a	H-4, H-5a, H-6a, H-6b, H-9
6a	1.21 (*dddd*, 5.3, 7.9, 8.5, 12.9)	30.5	H-5a, H-5b, H-6b, H-7	C-5, C-7, C-8, C-8a	H-5a, H-5b, H-6b, H-7, H-9
6b	1.44 (*dddd*, 4.0, 5.5, 5.9, 12.9)		H-5a, H-5b, H-6a, H-7	C-5, C-7, C-8, C-8a, C-9	H-5a, H-5b, H-6a, H-7, H-9
7	2.17 (*ddq*, 4,0, 8.5, 6.8)	22.2	H-6a, H-6b, H-9, H-10a, H-10b		H-5a, H-6a, H-6b, H-9, H-10a
8		144.2			
8a		146.6			
8b	5.24 (*dddd*, 0.5, 1.2, 2.2, 7.3)	84.2	H-3a, H-4, H-5a	C-2, C-8a	H-3a, H-10b
9	0.96 (*d*, 6.8)	18.9	H-7	C-5, C-6, C-8	H-5b, H-6a, H-6b, H-7, H-10a
10a	5.03 (*br*, *s*)	110.9	H-7, H-10b	C-4a, C-5, C-7, C-8	H-7, H-9, H-10b
10b	5.38 (*br*, *s*)		H-7, H-10a	C-4a, C-5, C-7, C-8	H-8b, H-10a
2’	5.02 (*dq*, 1.5, 1.5)	100.7	H-3’, H-7’	C-4’, C-5’, C-6’	H-3’, H-6’
3’	5.67 (*dq*, 1.5, 1.5)	140.6	H-2’, H-7’	C-2’, C-5’	H-2’, H-7’
4’		135.2			
5’		169.8			
6’	7.36 (*d*, 2.5)	151.4	H-3a	C-2, C-3, C-3a, C-2’	H-2’
7’	1.33 (*dd*, 1.5, 1.5)	10.2	H-2’, H-3’	C-2’, C-3’, C-4’, C-5’	H-3’
4-OH	1.11 (*s*)		H-4		

The authors apologize for this error and state that this does not change the scientific conclusions of the article in any way. The original article has been updated.

