# Supplementary material for: Identification of novel canonical strigolactones produced by tomato
Source: Front Plant Sci. 2022 Dec 14;13:1064378. doi: 10.3389/fpls.2022.1064378 (PMC9794758; doi:10.3389/fpls.2022.1064378)
Supplement: Supplementary file 1 [file DataSheet_1.docx]

Supplementary Material

# Supplementary Methods

*General procedure*

All reactions were carried out under Ar atmosphere in dry solvents unless otherwise stated. IR spectra were measured on Jasco FT/IR-4200 spectrometer (ATR). ^1^H NMR (400 MHz) and ^13^C NMR (100 MHz) data were recorded by Jeol JNM-ECS400. Chemical shifts (δ) were reported in ppm and referenced to the residual solvent peak as the internal standard (CDCl_3_: δ_H_ = 7.26, δ_C_ = 77.0). HRMS were recorded on SCIEX TripleTOF 5600. Column chromatography was performed on Kanto Chem. Co. Silica Gel 60N (particle size 63–210µm). Analytical thin layer chromatography (TLC) was carried out on Merck glass plates pre-coated with silica gel 60 F_254_ (0.25 mm).

*2,3,6-Trimethylphenyl trifluoromethanesulfonate (****SI-2****)*

To a solution of 2,3,6-trimethylphenol (**SI-1**, 2.00 g, 14.6 mmol), DMAP (35.8 mg, 0.293 mmol) and Et_3_N (3.10 mL, 22.0 mmol) in CH_2_Cl_2_ (50 mL) was added Tf_2_O (2.60 mL, 15.4 mmol) dropwise at 0 °C. After stirring at room temperature for 24 h, the reaction mixture was quenched with sat. NaHCO_3_ *aq.* and extracted with CH_2_Cl_2_. The combined organic layer was washed with sat. NH_4_Cl *aq.* and brine, dried over MgSO_4_, and concentrated *in vacuo.* The residue was purified by SiO_2_ column chromatography (*n-*hexane/EtOAc = 10:1) to afford **SI-2** (3.67 g, 13.6 mmol, 93%) as a colourless oil. FTIR (neat) νmax (cm^−1^); 1402, 1202, 1175, 1152, 1133, 1041, 938, 890, 809, 766, 727, 664, 620, 576, 557, 504; ^1^H NMR (400 MHz, CDCl_3_) δ 7.05 (1H, d, *J* = 7.6 Hz), 7.01 (1H, d, *J* = 7.6 Hz), 2.34 (3H, s), 2.27 (3H, s), 2.26 (3H, s); ^13^C NMR (100 MHz, CDCl_3_) δ 146.8, 137.1, 129.9, 129.2, 128.8, 128.5, 120.1, 19.9, 17.0, 13.6 ; HRMS (ESI^+^) *m/z* calced for C_10_H_11_F_3_NaO_3_S^+^ [M+Na]^+^ 291.0273; found, 291.0302.

*2-Allyl-1,3,4-trimethylbenzene (****SI-3****)*

To a solution of LiCl (474 mg, 11.1 mmol) in DMF (20 mL) were added allyltributyltin (1.38 mL, 4.46 mmol), **SI-2** (1.00 g, 3.72 mmol) and Pd(PPh_3_)_4_ (145 mg, 0.125 mmol) at room temperature. After stirring at 100 °C for 3 h, the reaction mixture was poured into sat. KF *aq*. and then kept stirring overnight at room temperature. The resulting mixture was extracted with Et_2_O, and the combined organic layer was washed with 1M NaOH *aq.* and brine, dried over MgSO_4_, and concentrated *in vacuo.* The residue was purified by SiO_2_ column chromatography (*n-*hexane/EtOAc = 30:1) to afford **SI-3** (432 mg, 2.69 mmol, 72%) as a colorless oil. FTIR (neat) νmax (cm^−1^); 2942, 1635, 1445, 991, 909, 803, 562; ^1^H NMR (400 MHz, CDCl_3_) δ 6.94 (1H, d, *J* = 8.0 Hz), 6.92 (1H, d, *J* = 8.0 Hz), 5.90 (1H, ddt, *J* = 16.8, 10.0, 5.6 Hz), 4.99 (1H, dq, *J* = 10.0, 2.0 Hz), 4.86 (1H, dq, *J* = 16.8, 2.0 Hz), 3.42 (2H, dt, *J* = 5.6, 2.0 Hz), 2.27 (3H, s), 2.26 (3H, s), 2.19 (3H, s); ^13^C NMR (100 MHz, CDCl_3_) δ 135.9, 135.5, 135.1, 134.2, 134.1, 127.6, 127.2, 114.8, 33.8, 20.7, 19.9, 15.3; HRMS (ESI^+^) *m/z* calced for C_12_H_16_Na^+^ [M+Na]^+^ 183.1144; found, 183.1161.

*3-(2,3,6-Trimethylphenyl)propane-1,2-diol (****SI-4****)*

To a solution of **SI-3** (403 mg, 2.51 mmol) in *t*-BuOH/H_2_O (7:1 v/v, 12.5 mL) were added NMO (618 mg, 5.28 mmol) and OsO_4_ in *t*-BuOH (0.078 M, 322 μL, 25 μmol) at 0 °C. After stirring at room temperature for 16 h, the reaction mixture was quenched with sat. Na_2_SO_3_ *aq.* and extracted with EtOAc. The combined organic layer was washed with brine, dried over Na_2_SO_4_, and concentrated *in vacuo.* The residue was passed through a short plug of silica gel (EtOAc) to afford **SI-4** (488 mg, quant.) as a colorless solid. Melting Point: 66–67 ℃; FTIR (film) νmax (cm^−1^); 3350 (br), 2921, 1458, 1024, 805, 537; ^1^H NMR (400 MHz, CDCl_3_) δ 6.96 (1H, d, *J* = 7.6 Hz), 6.93 (1H, d, *J* = 7.6 Hz), 3.95 (1H, m), 3.70 (1H, dd, *J* = 10.8, 1.2 Hz), 3.59 (1H, dd, *J* = 10.8, 7.2 Hz), 2.94 (1H, dd, *J* = 13.2, 9.2 Hz), 2.82 (1H, dd, *J* = 13.2, 4.8 Hz), 2.32 (3H, s), 2.25 (3H, s), 2.24 (3H, s), 2.04 (1H, br), 1.61 (1H, br); ^13^C NMR (100 MHz, CDCl_3_) δ 135.6, 134.78, 134.76, 134.2, 128.1, 127.7, 72.2, 66.3, 33.1, 20.8, 20.6, 16.0; HRMS (ESI^+^) *m/z* calced for C_12_H_18_NaO_2_^+^ [M+Na]^+^ 217.1199; found, 217.1200.

*2-(2,3,6-Trimethylphenyl)acetaldehyde (****SI-5****)*

To a solution of **SI-4** (488 mg, 2.51 mmol) in THF/H_2_O (3:1 v/v, 12.5 mL) was added NaIO_4_ (1.61 g, 7.54 mmol) at 0 °C. After stirring at 0 °C for 1 h, the reaction mixture was diluted with H_2_O and extracted with Et_2_O. The combined organic layer was washed with brine, dried over MgSO_4_, and concentrated *in vacuo.* The residue was passed through a short plug of silica gel (Et_2_O) to afford **SI-5** (384 mg, 2.37 mmol, 94%) as a colorless oil. FTIR (neat) νmax (cm^−1^); 2924, 1718, 1462, 1383, 1009, 806, 546, 519; ^1^H NMR (400 MHz, CDCl_3_) δ 9.67 (1H, t, *J* = 2.0 Hz), 7.02 (1H, d, *J* = 8.0 Hz), 6.98 (1H, d, *J* = 8.0 Hz), 3.79 (2H, d, *J* = 2.0 Hz), 2.27 (3H, s), 2.26 (3H, s), 2.18 (3H, s); ^13^C NMR (100 MHz, CDCl_3_) δ 199.3, 135.7, 134.7, 134.6, 129.1, 129.0, 127.6, 45.3, 20.7, 20.6, 16.0; HRMS (ESI^+^) *m/z* calced for C_11_H_14_NaO^+^ [M+Na]^+^ 185.0937; found, 185.0935.

*di-tert-Butyl (E)-2-(2,3,6-trimethylstyryl)malonate (****SI-6****)*

To a suspension of NaH (60%, 276 mg, 6.90 mmol) in THF (10 mL) was added di-*tert-*butyl malonate (1.50 mL, 6.90 mmol) at 0 °C. After stirring at room temperature for 10 min, **SI-5** (373 mg, 2.30 mmol) in THF (5 mL) was added to the mixture. After stirring for 15 h under reflux, the reaction mixture was quenched with sat. NH_4_Cl *aq.* and extracted with EtOAc. The combined organic layer was washed with brine, dried over MgSO_4_, and concentrated *in vacuo*. After removal of excess malonate by heating at 120 °C under vacuum, the residue was purified by SiO_2_ column chromatography (*n-*hexane/EtOAc = 30:1) to afford **SI-6** (277 mg, 0.768 mmol, 33%) as a yellow oil. FTIR (neat) νmax (cm^−1^); 2977, 1726, 1456, 1367, 1252, 1132, 970, 847, 806, 733; ^1^H NMR (400 MHz, CDCl_3_) δ 6.97 (1H, d, *J* = 7.6 Hz), 6.93 (1H, d, *J* = 7.6 Hz), 6.52 (1H, d, *J* = 16.8 Hz), 5.78 (1H, dd, *J* = 16.8, 8.8 Hz), 4.02 (1H, d, *J* = 8.8 Hz), 2.25 (6H, s), 2.21 (3H, s), 1.48 (18H, s); ^13^C NMR (100 MHz, CDCl_3_) δ 167.4, 136.5, 134.5, 134.4, 133.5, 132.9, 128.4, 127.2, 126.9, 81.7, 58.5, 27.9, 20.7, 20.3, 16.7; HRMS (ESI^+^) *m/z* calced for C_22_H_32_NaO_4_^+^ [M+Na]^+^ 383.2193; found, 383.2202.

*Aromatic CLA tert-butyl ester [tert-Butyl (2E,3E)-4-(2,3,6-trimethylphenyl)-2-((4-methyl-5-oxo-2,5-dihydrofuran-2-yloxy)methylene-1-yl)but-3-enoate] (****SI-7****)*

To a solution of **SI-6** (136 mg, 377 μmol) in CH_2_Cl_2_ (3.8 mL) was added DIBAL (1.02 M in hexane, 740 µL, 755 µmol) at −78 °C. After stirring at −78 °C for 1 h, the reaction mixture was quenched by the addition of MeOH and then sat. Rochelle salt *aq*. After stirring at room temperature for 2 h, the resulting mixture was extracted with CH_2_Cl_2_. The combined organic layer was washed with sat. Rochelle salt *aq.*, dried over MgSO_4_, and concentrated *in vacuo* to give the crude product (117 mg) as a yellow oil.

To a solution of the crude product (117 mg) in THF (2.0 mL) was added *t*-BuOK (42.3 mg, 377 μmol) at 0 °C. After stirring at 0 °C for 10 min, a solution of 4-bromo-2-methyl-2-buten-4-olide (66.7 mg, 377 μmol) in THF (0.5 mL) was added. After stirring at 0 °C for 1 h, the reaction mixture was quenched with sat. NH_4_Cl *aq.* (4.0 mL) and extracted with EtOAc. The combined organic layer was washed with H_2_O, dried over MgSO_4_, and concentrated *in vacuo*. The residue was purified by SiO_2_ column chromatography (hexane/CH_2_Cl_2_ = 1:2) to give **SI-7** (50.7 mg, 33% in 2 steps) as a yellow oil. FTIR (film) νmax (cm^−1^); ^1^H NMR (400 MHz, CDCl_3_) δ 7.50 (1H, s), 7.19 (1H, d, *J* = 16.8 Hz), 6.96 (1H, d, *J* = 8.0 Hz), 6.94 (1H, d, *J* = 8.0 Hz), 6.93 (1H, t, *J* = 1.6 Hz), 6.24 (1H, d, *J* = 16.8 Hz), 6.15 (1H, t, *J* = 1.6 Hz), 2.26 (3H, s), 2.25 (3H, s), 2.22 (3H, s), 2.00 (3H, t, *J* = 1.6 Hz), 1.53 (9H, s); ^13^C NMR (100 MHz, CDCl_3_) δ 170.6, 165.7, 151.7, 141.2, 138.2, 135.4, 134.3, 134.1, 133.3, 132.2, 128.1, 127.0, 123.5, 114.3, 100.4, 81.0, 28.3, 20.9, 20.4, 17.0, 10.6; HRMS (ESI^+^) *m/z* calced for C_23_H_28_NaO_5_^+^ [M+Na]^+^ 407.1829; found, 407.1842.

*Aromatic CLA [rac-(2E,3E)-4-(2,3,6-trimethylphenyl)-2-((4-methyl-5-oxo-2,5-dihydrofuran-2-yloxy)methylene-1-yl)but-3-enoic acid] (****SI-8****)*

To a solution of **SI-7** (36.9 mg, 96.0 μmol) in CH_2_Cl_2_ (1.0 mL) were added 2,6-lutidine (33 μL, 288 μmol) and TESOTf (88 μL, 384 μmol) at 0 °C. After stirring at 0 °C for 30 min, the reaction mixture was quenched with sat. NH_4_Cl *aq.* and extracted with CH_2_Cl_2_. The combined organic phase was washed with H_2_O, dried over MgSO_4_, and concentrated *in vacuo*. The residue was purified by SiO_2_ column chromatography (hexane/EtOAc = 1:1, containing 0.5% AcOH) to give **SI-8** (9.0 mg, 29%) as a colorless amorphous solid. FTIR (neat) νmax (cm^−1^); ^1^H NMR (400 MHz, CDCl_3_) δ 7.74 (1H, s), 7.29 (1H, d, *J* = 16.8 Hz), 6,92–6.99 (3H, m), 6.26 (1H, d, *J* = 16.8 Hz), 6.20 (1H, t, *J* = 1.6 Hz), 2.25 (6H, s), 2.21 (3H, s), 2.02 (3H, t, *J* = 1.6 Hz); ^13^C NMR (100 MHz, CDCl_3_) δ 170.9, 170.3, 153.9, 141.0, 137.9, 135.7, 134.3, 134.2, 133.3, 133.1, 128.2, 127.1, 122.5, 111.7, 100.4, 20.9, 20.4, 17.0, 10.7; HRMS (ESI^+^) *m/z* calced for C_19_H_21_O_5_^+^ [M+H]^+^ 329.1384; found, 329.1388.

# Supplementary Data

## Supplementary Figures


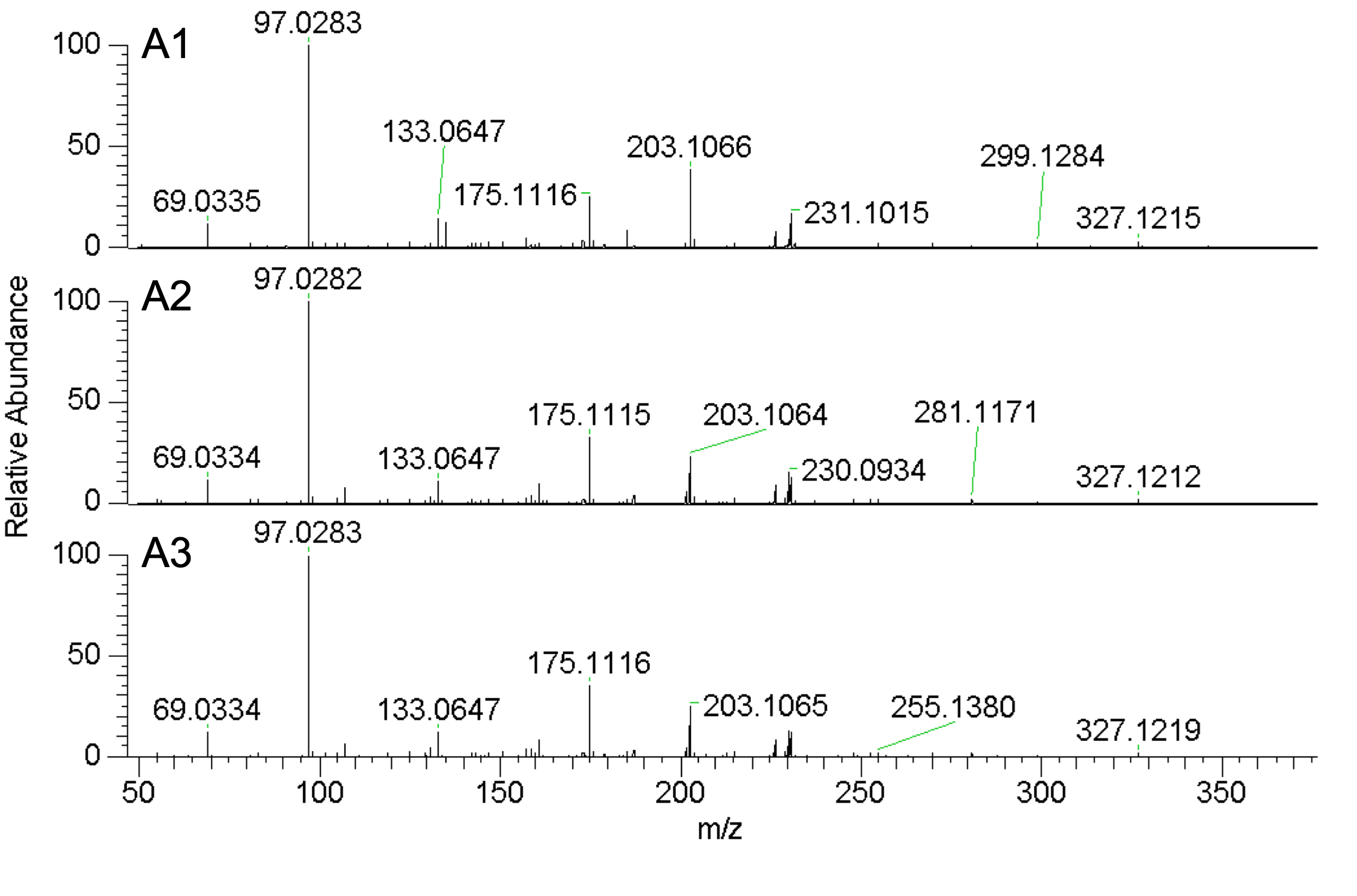


**Supplementary Figure S1.** HR-ESI-MS spectra of A1, A2, and A3.

**Supplementary Figure S2.** CD spectra of A1, A2, and A3.


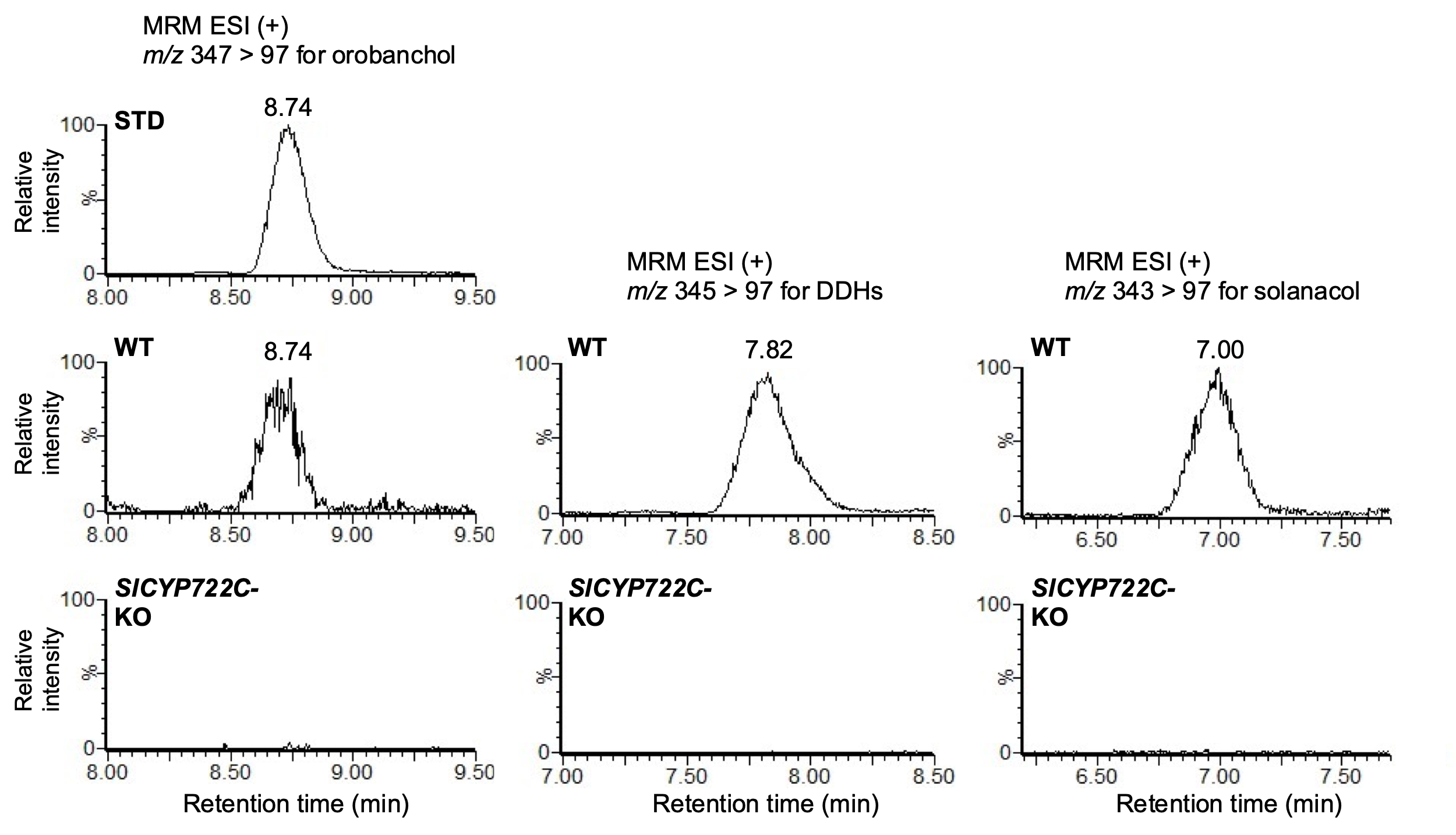


**Supplementary Figure S3.** The multiple reaction monitoring (MRM) chromatogram of orobanchol, DDHs, and solanacol in tomato root exudates of wild-type (WT) and *SlCYP722C*-KO plants.


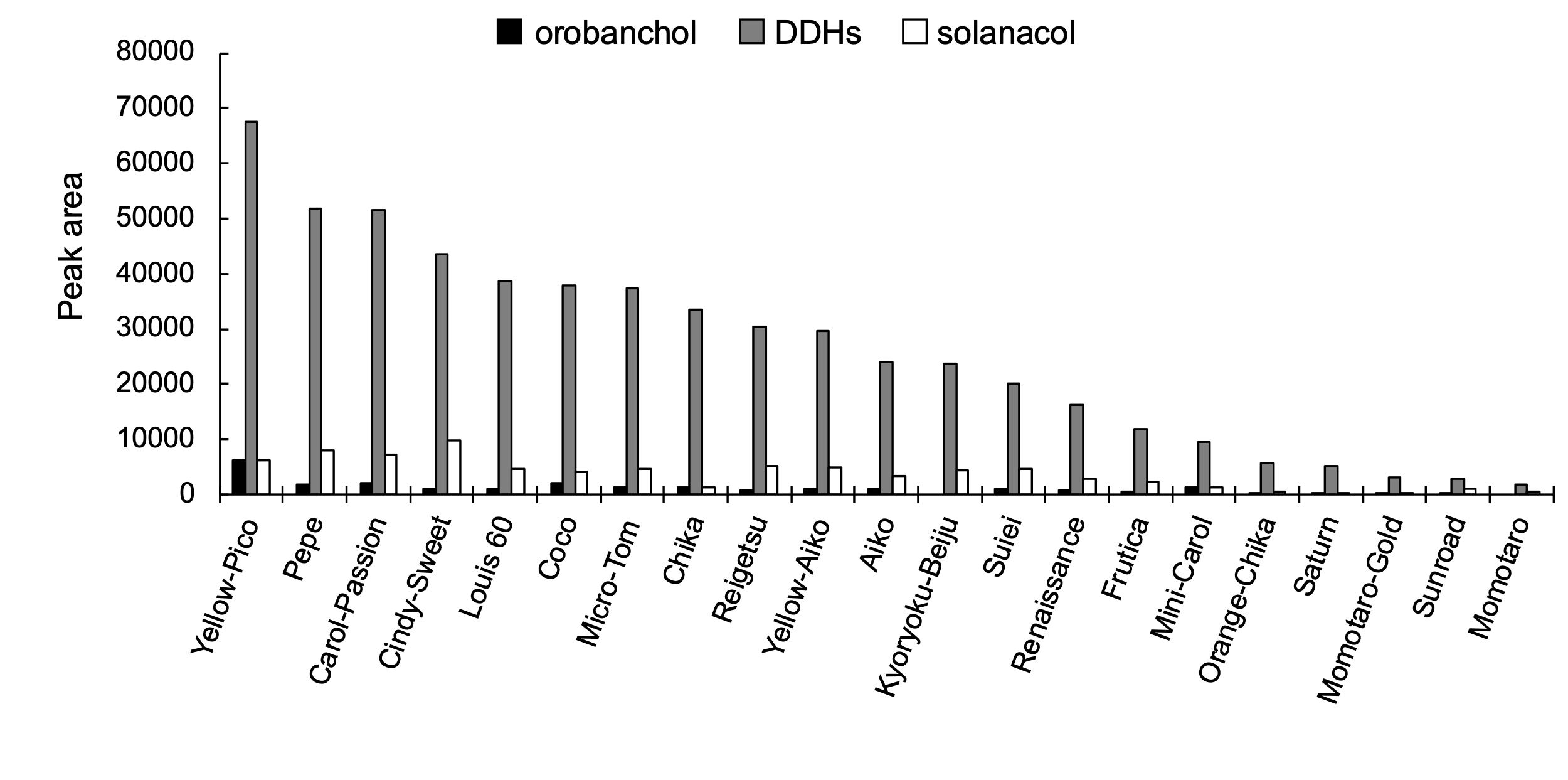


**Supplementary Figure S4.** Qualitative and quantitative analysis of SLs in various tomato cultivars.

**Supplementary Figure S5.** ^1^H-NMR spectrum of 6,7-didehydroorobanchol/A1.

**Supplementary Figure S6.** ^1^H-^1^H COSY spectrum of 6,7-didehydroorobanchol/A1.

**Supplementary Figure S7.** NOESY spectrum of 6,7-didehydroorobanchol/A1.

**Supplementary Figure S8.** ^1^H-NMR spectrum of phelipanchol/A2.

**Supplementary Figure S9.** ^13^C-NMR spectrum of phelipanchol/A2.

**Supplementary Figure S10.** ^1^H-^1^H COSY spectrum of phelipanchol/A2.

**Supplementary Figure S11.** HSQC spectrum of phelipanchol/A2.

**Supplementary Figure S12.** NOESY spectrum of phelipanchol/A2.

**Supplementary Figure S13.** HMBC spectrum of phelipanchol/A2.

**Supplementary Figure S14.** ^1^H-NMR spectrum of epiphelipanchol/A3.

**Supplementary Figure S15.** ^1^H-^1^H COSY spectrum of epiphelipanchol/A3.

**Supplementary Figure S16.** NOESY spectrum of epiphelipanchol/A3.


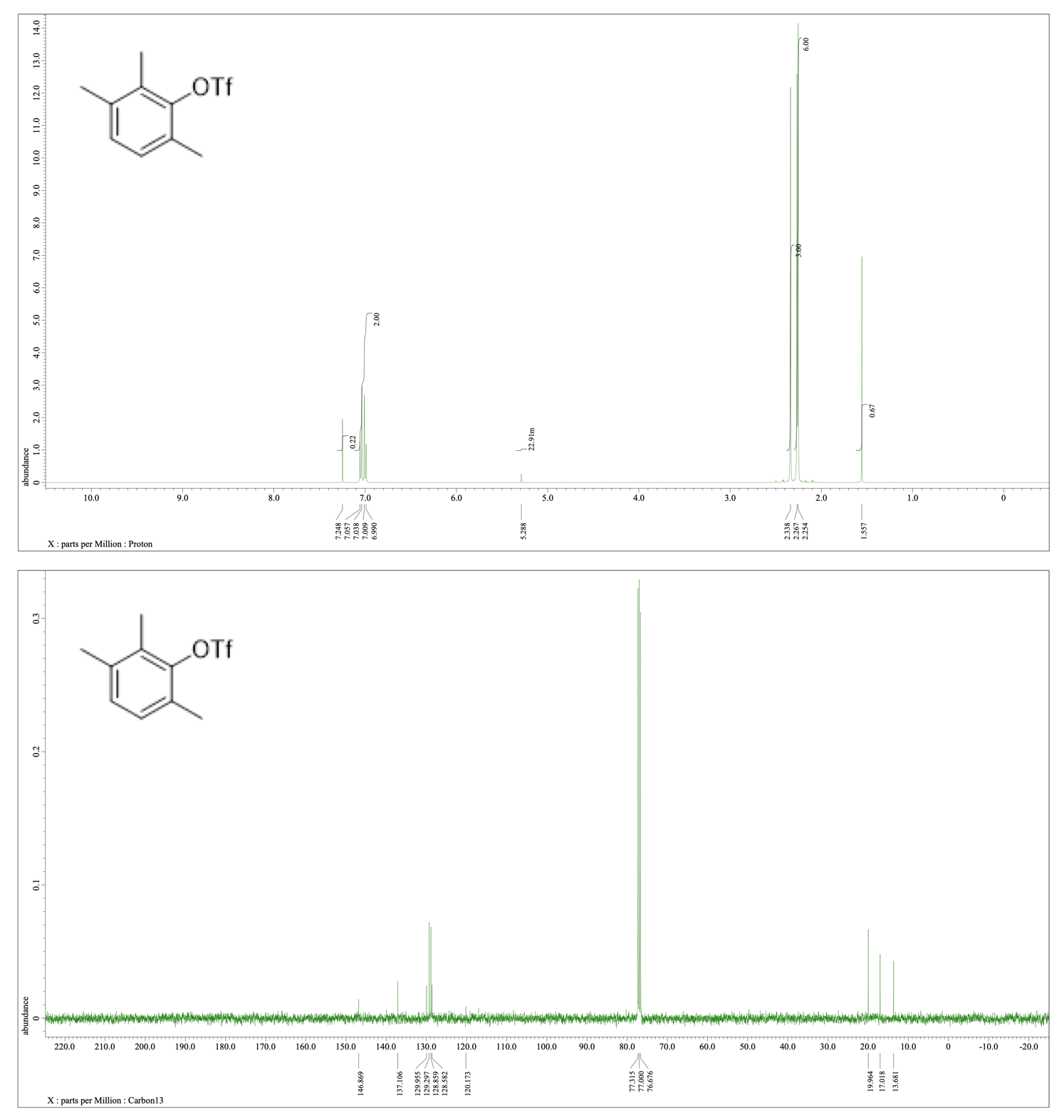


**Supplementary Figure S17.** ^1^H- and ^13^C-NMR spectra of **SI-2**.


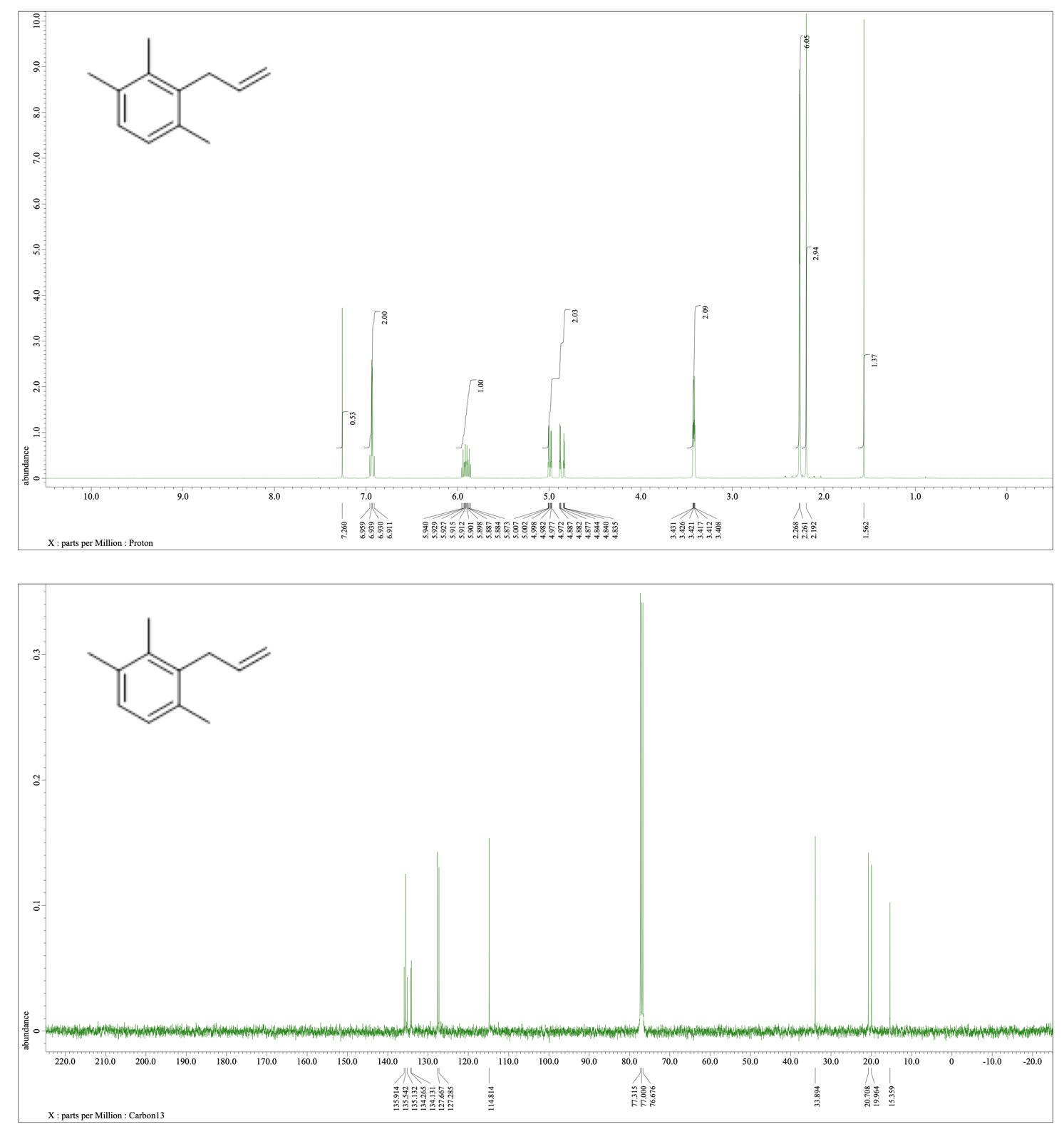


**Supplementary Figure S18.** ^1^H- and ^13^C-NMR spectra of **SI-3**.


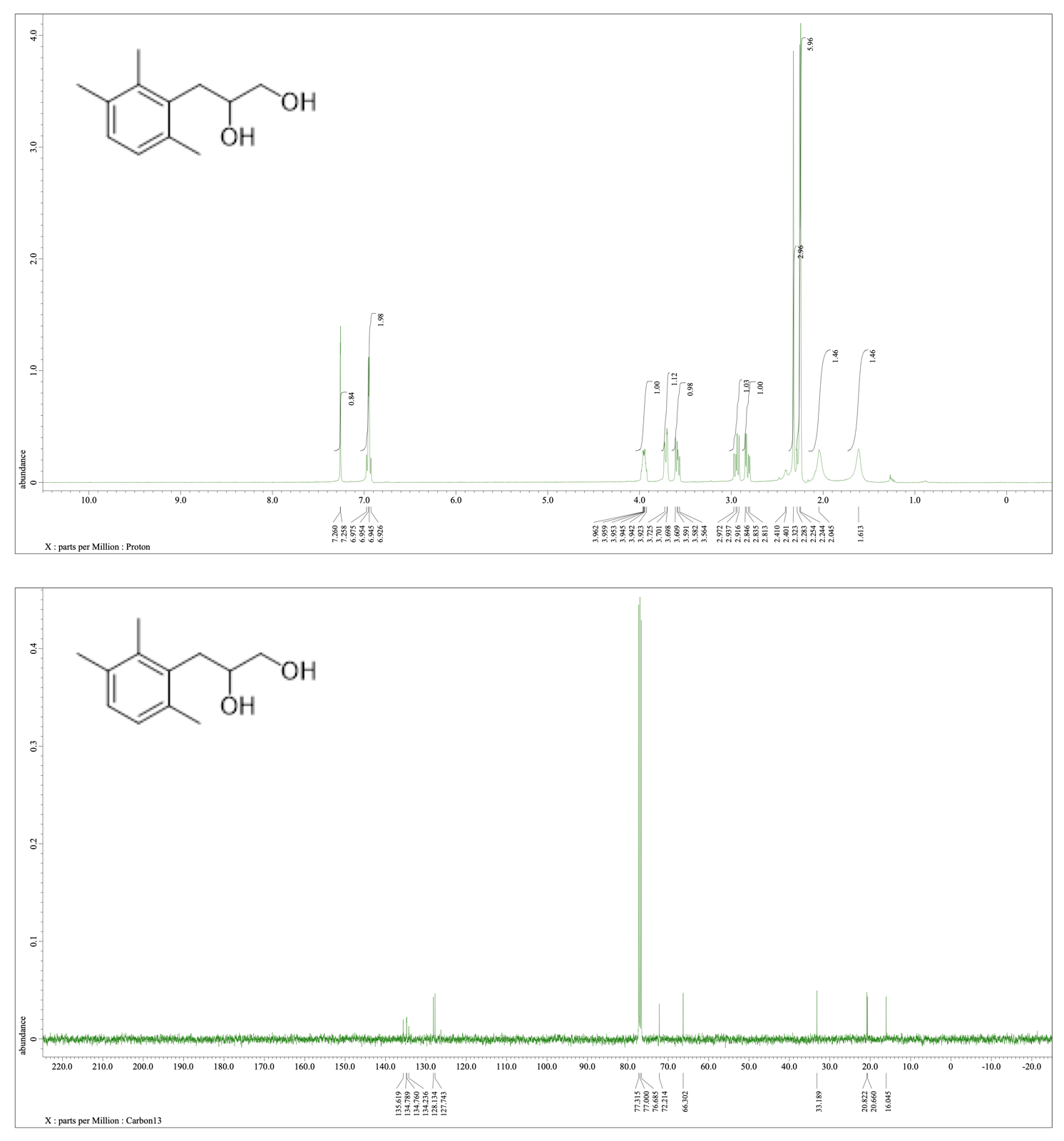


**Supplementary Figure S19.** ^1^H- and ^13^C-NMR spectra of **SI-4**.


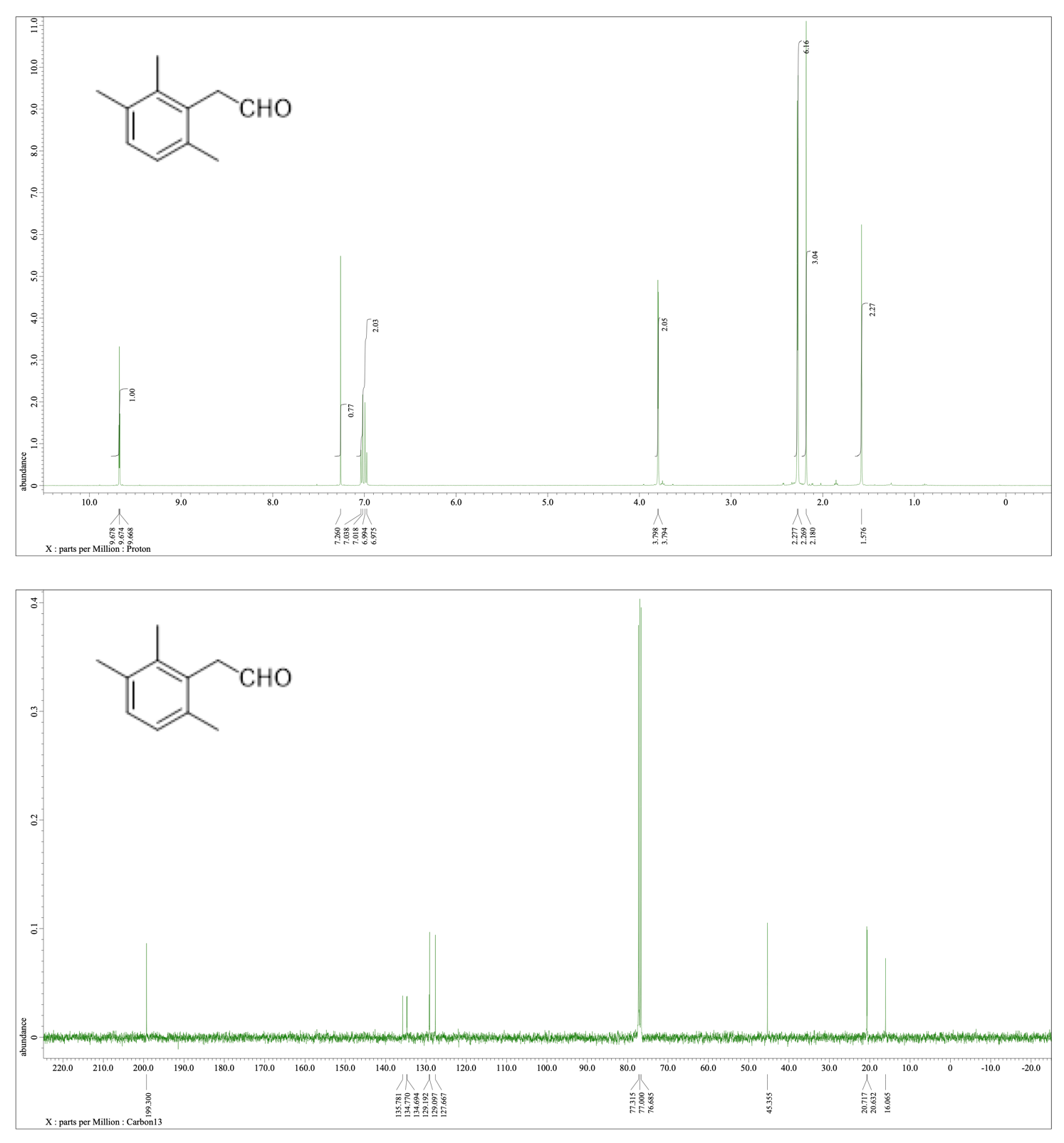


**Supplementary Figure S20.** ^1^H- and ^13^C-NMR spectra of **SI-5**.


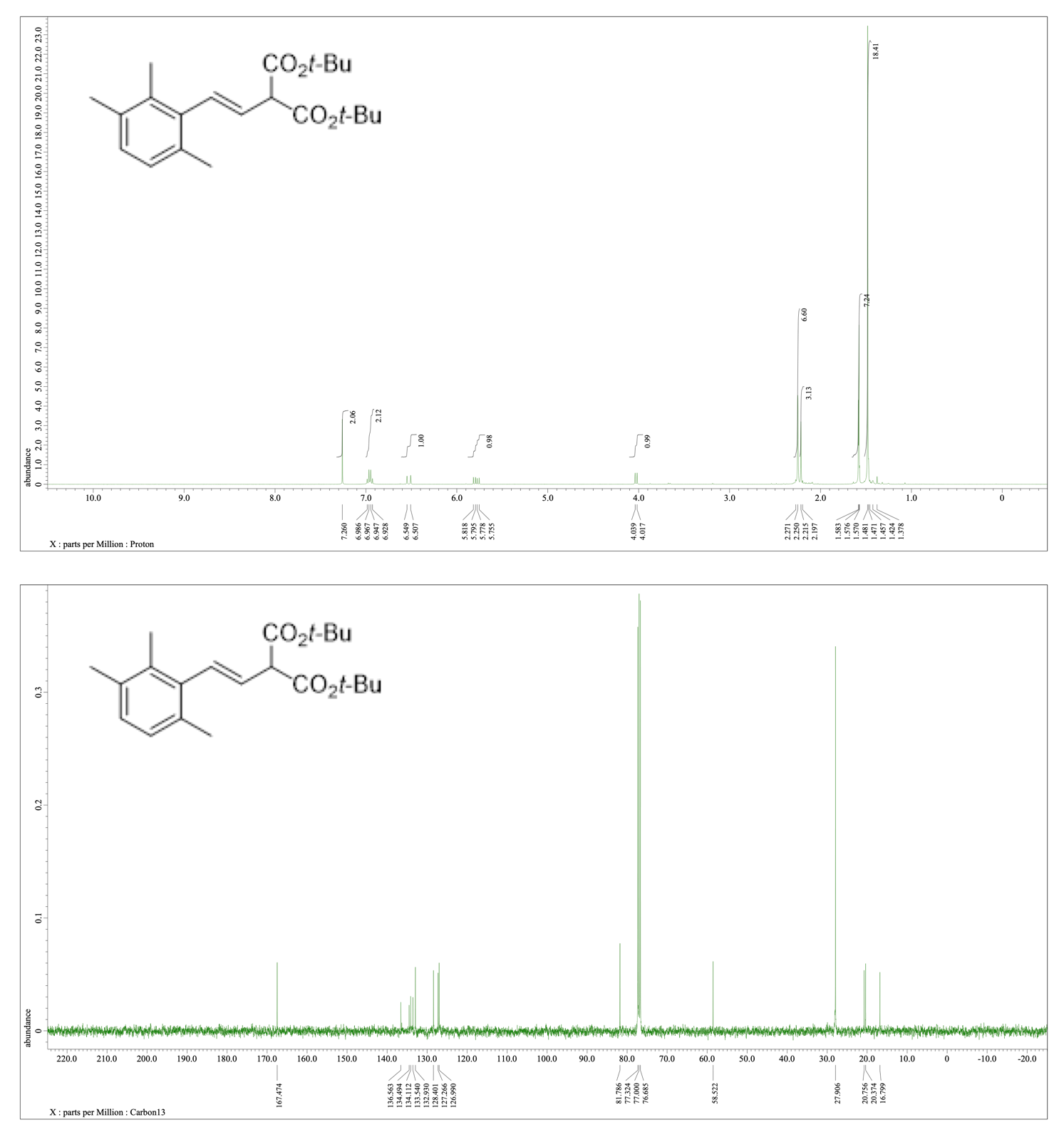


**Supplementary Figure S21.** ^1^H- and ^13^C-NMR spectra of **SI-6**.


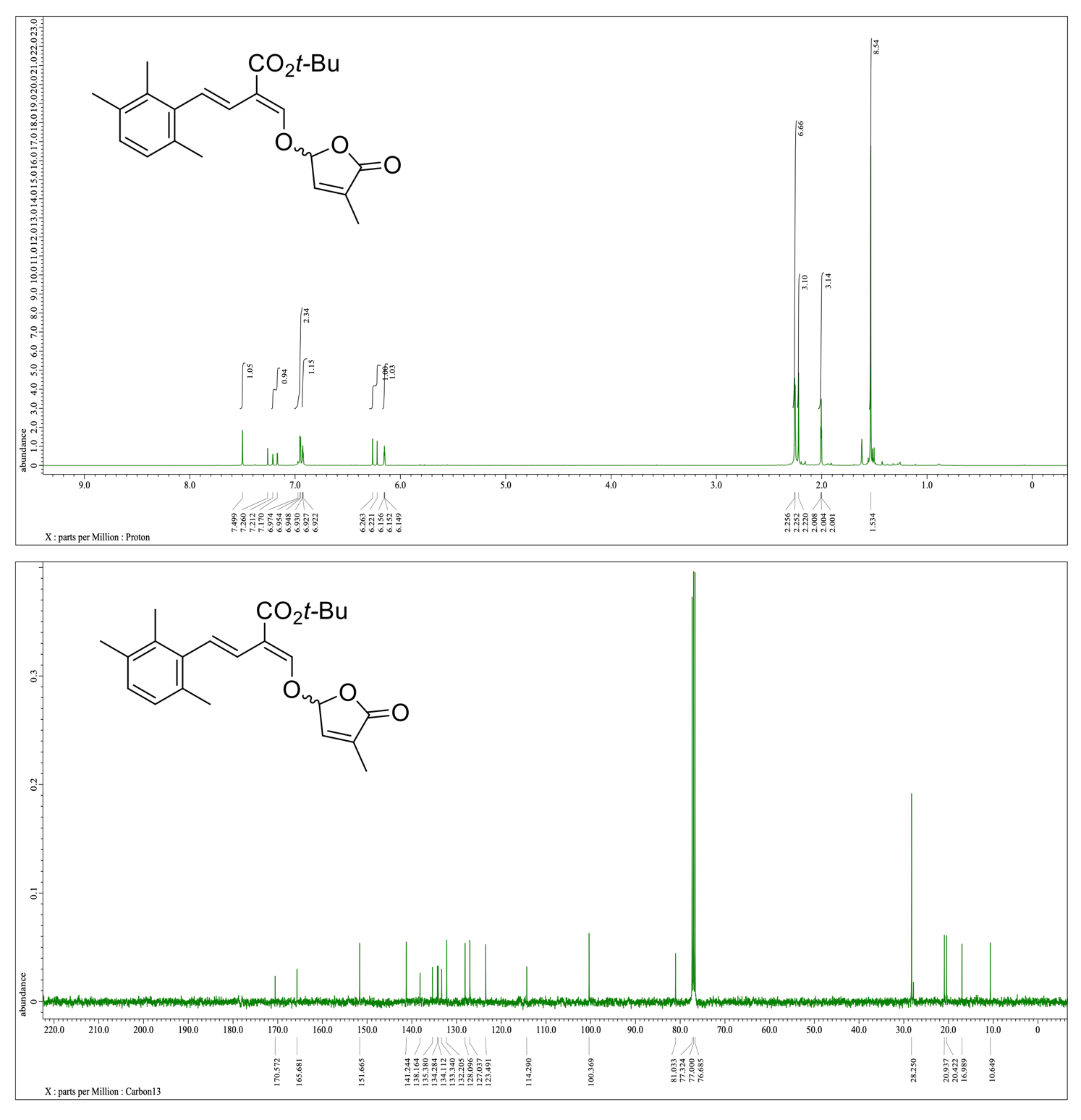


**Supplementary Figure S22.** ^1^H- and ^13^C-NMR spectra of **SI-7**.


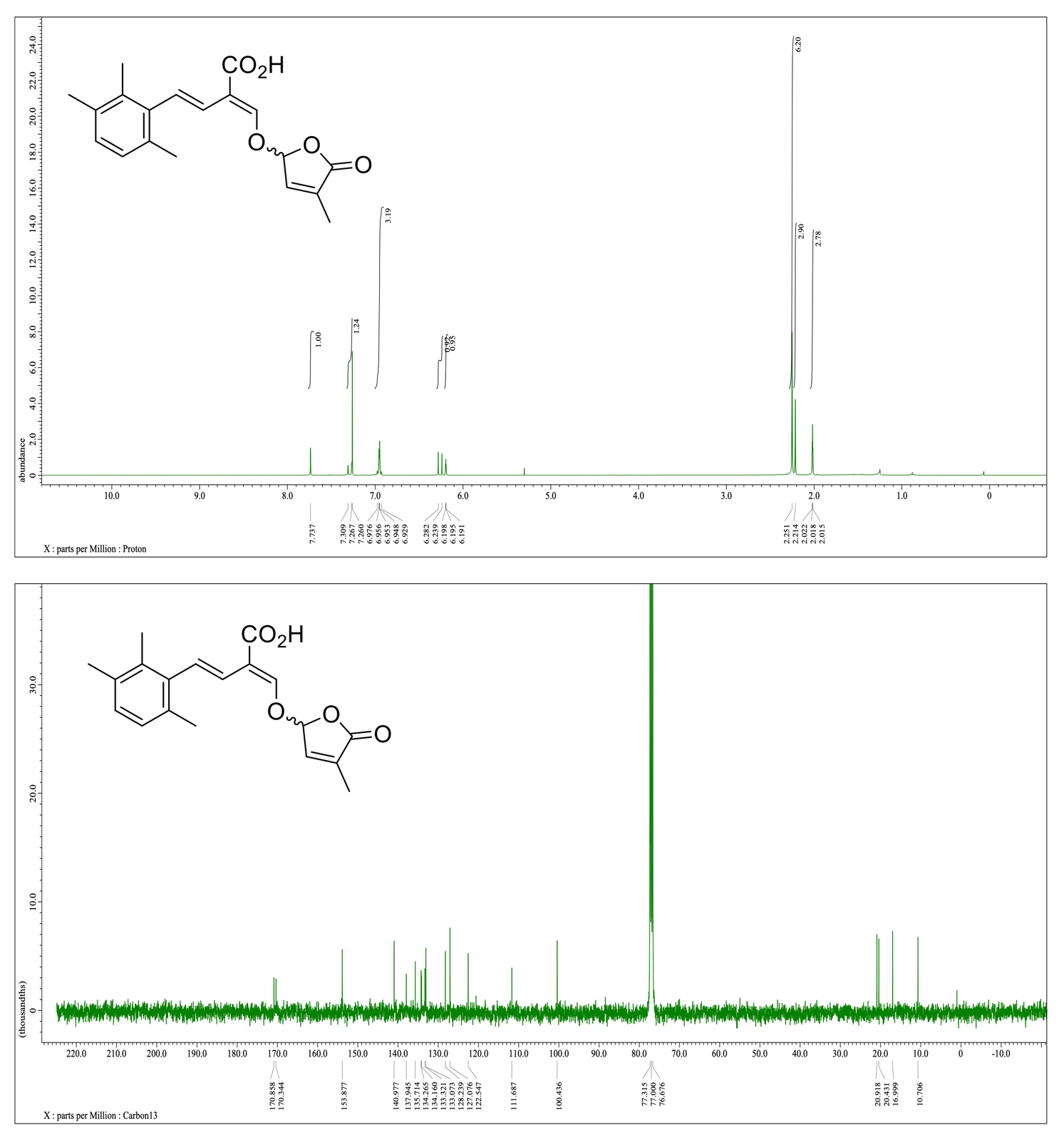


**Supplementary Figure S23.** ^1^H- and ^13^C-NMR spectra of **SI-8** (aromatic CLA).

## Supplementary Tables

**Supplementary Table S1.** NMR spectroscopic data of A3.

| No. | δ ^1^H (mult., *J* Hz) | ^1^H-^1^H COSY | NOESY |
| --- | --- | --- | --- |
| 2 |  |  |  |
| 3 |  |  |  |
| 3a | 3.18 (*ddd*, 2.0, 2.5, 7.3) | H-4, H-8b, H-6' | H-8b |
| 4 | 4.33 (*br*, *s*) | H-3a, H-8b, 4-OH |  |
| 4a |  |  |  |
| 5a | 1.98 (*dddd*, 1.9, 5.3, 5.6, 18.1) | H-5b, H-6a, H-6b, H-8b, H-10a | H-5b, H-6a, H-9 |
| 5b | 1.60 (*ddd*, 5.6, 8.3, 18.1) | H-5a, H-6a, H-6b | H-5a |
| 6a | 1.17 (*dddd*, 5.3, 8.3, 8.9, 12,9) | H-5a, H-5b, H-6b, H-7 | H-5a, H-6b, H-9 |
| 6b | 1.45 (*dddd*, 3.9, 5.6, 5.6, 12.9) | H-5a, H-5b, H-6a, H-7 | H-6a, H-7 |
| 7 | 2.15 (*ddq*, 3.9, 8.9, 6.8) | H-6a, H-6b, H-9 | H-6b, H-9, H-10a |
| 8 |  |  |  |
| 8a |  |  |  |
| 8b | 5.29 (*ddd*, 1.9, 3.0, 7.3) | H-3a, H-4, H-5a | H-3a |
| 9 | 0.97 (*d*, 6.8) | H-7 | H-5a, H-6a, H-7, H-10a |
| 10a | 5.03 (*br*, *s*) | H-5a, H-10b | H-7, H-9, H-10b |
| 10b | 5.35 (*br*, *s*) | H-10a | H-10a |
| 2' | 4.98 (*dq*, 1.7, 1.5) | H-3', H-7' | H-6' |
| 3' | 5.66 (*dq*, 1.7, 1.5) | H-2', H-7' |  |
| 4' |  |  |  |
| 5' |  |  |  |
| 6' | 7.35 (*br*, *s*) | H-3a | H-2' |
| 7' | 1.33 (*dd*, 1.5, 1.5) | H-2', H-3' |  |
| 4-OH | 1.07 (*d*, 5.5) | H-4 |  |

**Supplementary Table S2.** NMR spectroscopic data of A1.

| No. | δ ^1^H (mult., *J* Hz) | ^1^H-^1^H COSY | NOESY |
| --- | --- | --- | --- |
| 2 |  |  |  |
| 3 |  |  |  |
| 3a | 3.17 (*ddd*, 2.0, 2.5, 7.3) | H-8b, H-6' | H-8b |
| 4 | 4.26 (*br*, *s*) | H-8b |  |
| 4a |  |  |  |
| 5a | 2.47 (*dddd*, 1.0, 1.7, 3.0, 22.6) | H-5b, H-6, H-7, H-8b | H-5b, H-6 |
| 5b | 2.12 (*dddd*, 1.5, 2.3, 3.2, 22.6) | H-5a, H-6, H-7 | H-5a, H-6 |
| 6a | 5.40 (*ddd* 3.0, 3.2, 9.9) | H-5a, H-5b, H-7 | H-5a, H-5b |
| 6b |  |  |  |
| 7 | 5.34 (*ddd*, 1.5, 1.7, 9.9) | H-5a, H-5b, H-6 | H-9, H-10 |
| 8 |  |  |  |
| 8a |  |  |  |
| 8b | 5.22 (*dddd*, 1.0, 2.0, 2.3, 7.3) | H-3a, H-4, H-5a | H-3a, H-9 |
| 9 | 1.06 (*s*) |  | H-7, H-8b, H-10 |
| 10a | 1.30 (*s*) |  | H-7, H-9 |
| 10b |  |  |  |
| 2' | 5.00 (*dq*, 1.8, 1.5) | H-3', H-7' | H-3', H-6' |
| 3' | 5.66 (*dq*, 1.8, 1.5) | H-2', H-7' | H-2' |
| 4' |  |  |  |
| 5' |  |  |  |
| 6' | 7.37 (*d*, 2.5) | H-3a | H-2' |
| 7' | 1.33 (*dd*, 1.5, 1.5) | H-2', H-3' |  |
| 4-OH |  |  |  |
